# Directed Synthesis of Gold Nanoparticle Superstructures Using Self-Assembling Peptoids Containing Metal-Bonding N‑Heterocyclic Carbenes

**DOI:** 10.1021/acs.nanolett.5c02998

**Published:** 2025-07-11

**Authors:** Lingcong Ge, Thi Kim Hoang Trinh, Changning Li, Florian Mayer, Jia Min Chin, Chun-Long Chen, Michael R. Reithofer

**Affiliations:** † Institute of Inorganic Chemistry, Faculty of Chemistry, 27258 University of Vienna, Währinger Straße 42, Vienna 1090, Austria; ‡ Physical Sciences Division, 6865 Pacific Northwest National Laboratory, Richland, Washington 99352, United States; § Department of Chemical Engineering, University of Washington, Seattle, Washington 98195, United States; ∥ Institute of Materials Chemistry and Research, Faculty of Chemistry, 31285 University of Vienna, Währinger Straße 42, Vienna 1090, Austria; ⊥ Department of Functional Materials and Catalysis, Faculty of Chemistry, University of Vienna, Währinger Straße 42, Vienna 1090, Austria; # Vienna Doctoral School in Chemistry (DoSChem), University of Vienna, Währinger Str. 42, 1090 Vienna, Austria

**Keywords:** N-heterocyclic carbenes, gold nanoparticle superstructures, self-assembling peptoids, organic−inorganic nanohybrid, nanoparticle vesicles

## Abstract

N-Heterocyclic carbene (NHC) ligands, with strong metal-binding affinity, offer a robust platform for constructing organic–inorganic nanohybrids with high stability and tunable properties. However, achieving precise structural control in a simple manner remains challenging. Here, we report a one-pot synthesis of nanohybrids using self-assembling peptoids functionalized with histidine-2-ylidene, which simultaneously enable peptoid assembly and NHC–metal binding. Histidine-2-ylidene-functionalized peptoids were designed to self-assemble and provide NHC binding sites, while AuNPs served as the inorganic component due to NHCs’ strong affinity for gold surfaces. The resulting peptoid-NHC@AuNPs form well-defined vesicles that are characterized by UV–vis spectroscopy, X-ray photoelectron spectroscopy, and electron microscopy. Importantly, the vesicle size and morphology can be tuned via the peptoid sequence or environmental conditions. Further experiments highlight the crucial role of the NHC sites in the formation and stabilization of these nanohybrids. This modular strategy offers a versatile route to fabricating functional NHC-based nanohybrids for potential applications in sensing.

## Graphical abstract

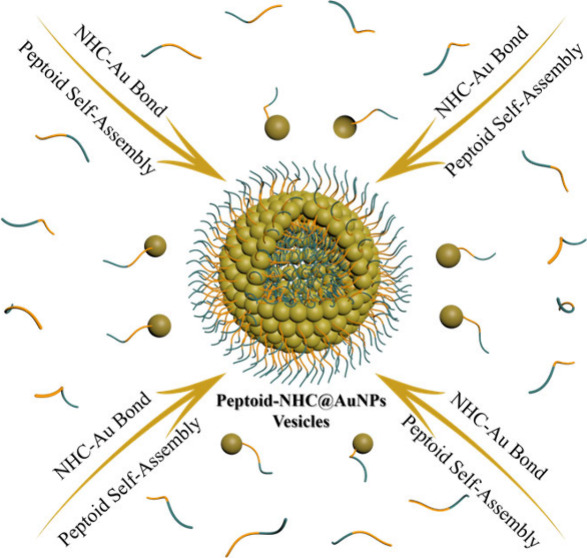

N-Heterocyclic carbenes (NHCs) are gaining attention as persistent surface ligands for stabilizing and functionalizing metal nanoparticles (MNPs).^1,2^ Their strong σ-donating ability, combined with electron-withdrawing and π-donating effects from adjacent nitrogen atoms, makes them ideal for MNP functionalization.^3,4^ Further, NHCs offer synthetic flexibility, enabling precise control over steric and electronic properties.^2,5,6^ For instance, Fery et al. used NHCs to anchor redox-active triphenylamines onto AuNPs, enhancing their electrochromic performance and stability.^7^ Similarly, our group developed Peg-NHC@AuNPs with azide functionalities, demonstrating exceptional colloidal stability and biorthogonal click reactivity.^8^

Nanohybrids, integrating organic and inorganic components, display distinctive morphological, chemical, and physical properties.^9^ The synthetic flexibility of NHCs, coupled with their strong metal surface affinity, enables the construction of NHC-linked nanohybrids with enhanced stability, tunability, and synergistic properties.^10–12^ Current NHC-modified nanohybrids are mainly limited to polymer-NHC-NPs,^10^ MOF-NHC-NPs,^13^ and rGO-NHC-NPs,^14^ with restricted control over nanoparticle integration and application. For instance, polymer-NHC-NPs are typically synthesized via polymer functionalization with molecular gold compounds followed by reduction. Nazemi et al. reported a bottom-up approach for polymeric mesoionic NHC–Au­(I) complexes, yielding water-soluble AuNPs.^10^ Our group recently developed NHC–metal bonds between hyper-cross-linked polymers and AuNPs, achieving exceptional stability and recyclability in catalysis.^15^ However, the currently reported methods often result in random and irregular MNP distribution throughout the composite, which limits the wide application of NHC-linked nanohybrids.

Molecular self-assembly is a promising strategy for fabricating nanohybrids with controlled hierarchical structures.^9^ While molecular self-assembly has been successfully applied to organic–inorganic nanohybrids, achieving rational design remains challenging due to its complexity.^9,16,17^ Peptoids, sequence-defined glycine polymers with N-substituted side chains, offer molecular programmability for predictable structures and functions, making them valuable organic components in nanohybrid systems.^18–20^ Peptoid-based methods for functional nanohybrid synthesis have emerged as a unique strategy due to their biocompatibility, high stability, and peptide-/protein-like molecular recognition capabilities.^21–23^ In addition, peptoid-mediated nanoparticle synthesis and self-assembly have yielded diverse nanohybrids with tunable interactions and crystal facet expression.^24–28^ However, despite advances in peptoid–inorganic interfaces^24,21,29^ and peptoid-based hierarchical self-assembly^16,18,19^ and the promise of NHCs in the MNP synthesis, NHCs have never been used to combine with well-controlled peptoid self-assembly for the synthesis and assembly of MNPs to develop functional organic–inorganic hybrids.^16,19,20^

In this study, we propose a one-pot approach for fabricating organic–inorganic nanohybrids based on the coordinative attachment of metal-binding NHCs into self-assembling peptoids and their self-assembly into hierarchical structures combined with simultaneous AuNP formation and assembly. AuNPs are attractive as representative inorganic components due to their good stability, low cytotoxicity, and distinctive optical properties, and AuNP-based hybrid materials show potential in sensing, catalytic, and biomedical applications.^30–32^ In addition, the high binding energy of NHC to the Au(111) surface makes it a promising linkage between peptoid and AuNPs.^33–35^ A series of histidin-2-ylidene-modified peptoids with different hydrophobicities and flexibilities were synthesized for the directed formation of peptoid-NHC@AuNPs vesicles. The successful synthesis of close-packed AuNP superlattices within peptoid-NHC@AuNPs vesicles was confirmed by UV–vis spectroscopy and high-resolution transmission electron microscopy (HRTEM), and the formation of NHC–Au bonds was verified by X-ray photoelectron spectroscopy (XPS). Furthermore, a systematic study of the peptoid-NHC@AuNP vesicles was conducted based on EM characterization and critical micelle concentration (CMC) analysis, elucidating that such structures arise from a cooperative interplay between the inherent self-assembly propensity of peptoids and the NHC sites, which serve as effective binding sites for the integration of AuNPs. To further demonstrate the critical role of having robust NHC–Au bonds in the formation of these Au NP vesicles, a comparative study with a thiol-based peptoid was conducted, demonstrating the enhanced resistance of our NHC-based vesicles against exogenous thiols. In addition, this peptoid-NHC@AuNP system exhibits enhanced Raman scattering activity, indicating its potential as a hot spot engineering approach.

In order to utilize histidin-2-ylidene as a building block in peptoid synthesis, histidin-2-ylidene bearing a free N-terminus was obtained via an alkylation reaction using methyl iodine starting from Nα-Boc-l-histidine. Subsequent deprotection of the Boc group was achieved in a 5% trifluoroacetic acid/dichloromethane solution, and an ion exchange was performed to obtain the final product as an HCl salt (SI1 for experimental details, Figures S1 and S2). Subsequently, histidin-2-ylidene-functionalized peptoids were obtained via standard solid phase synthesis (SI1 for experimental details). As peptoid hydrophobicity has been proven to be an important factor for assembling vesicular structures, we developed a series of peptoids with different hydrophobicities and flexibility. These peptoids included benzene rings sourced from *N*-(phenylmethyl)­glycine (**Npm**) or *N*-(2-phenylethyl)­glycine (**Npe**) within the hydrophobic domain, alongside polar fragments such as *N*-(2-methoxyethyl)­glycine (**Nome**) and diglycolic acid (**Dig**) (Scheme 1, Figures S3–S6).^36,37^

**Scheme 1:**
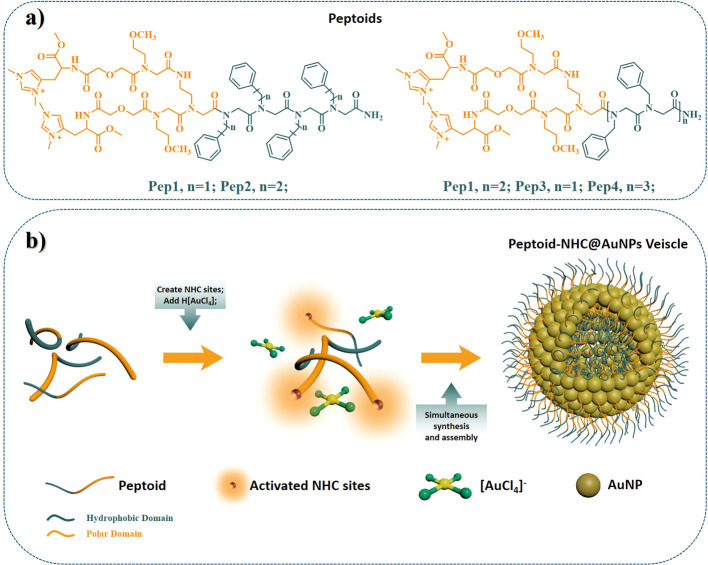
(a) Chemical Structure of Peptoids Utilized in the Study; (b) Schematic Representation of Peptoid-NHC@AuNPs Vesicle Formation with Simultaneous NP Synthesis and Assembly

The general synthesis for peptoid-NHC@AuNPs vesicles is illustrated in Scheme 1 and Chart S1, using the starting peptoids Npm_4_(NomeDig-His)_2_ (**Pep1**) and Npe_4_(NomeDig-His)_2_ (**Pep2**). Notably, NaH was used to create free NHC sites, followed by the addition of H­[AuCl_4_]. Subsequently, NaBH_4_ was utilized as a reducing agent to obtain the final peptoid-NHC@AuNPs compound (SI1 for the experimental details). The successful NP formation can be observed through an instantaneous color change of the peptoid-Au mixture from pale yellow to dark red (Figure 1a).^38^ To further confirm the formation of gold nanoparticles, UV–vis spectroscopy was employed. Typical localized surface plasmon resonance (LSPR) peaks were observed at 528 nm for **Pep1-NHC@AuNPs** and 522 nm for **Pep2-NHC@AuNPs** in toluene (Figure 1a).^39^

**Figure 1:**
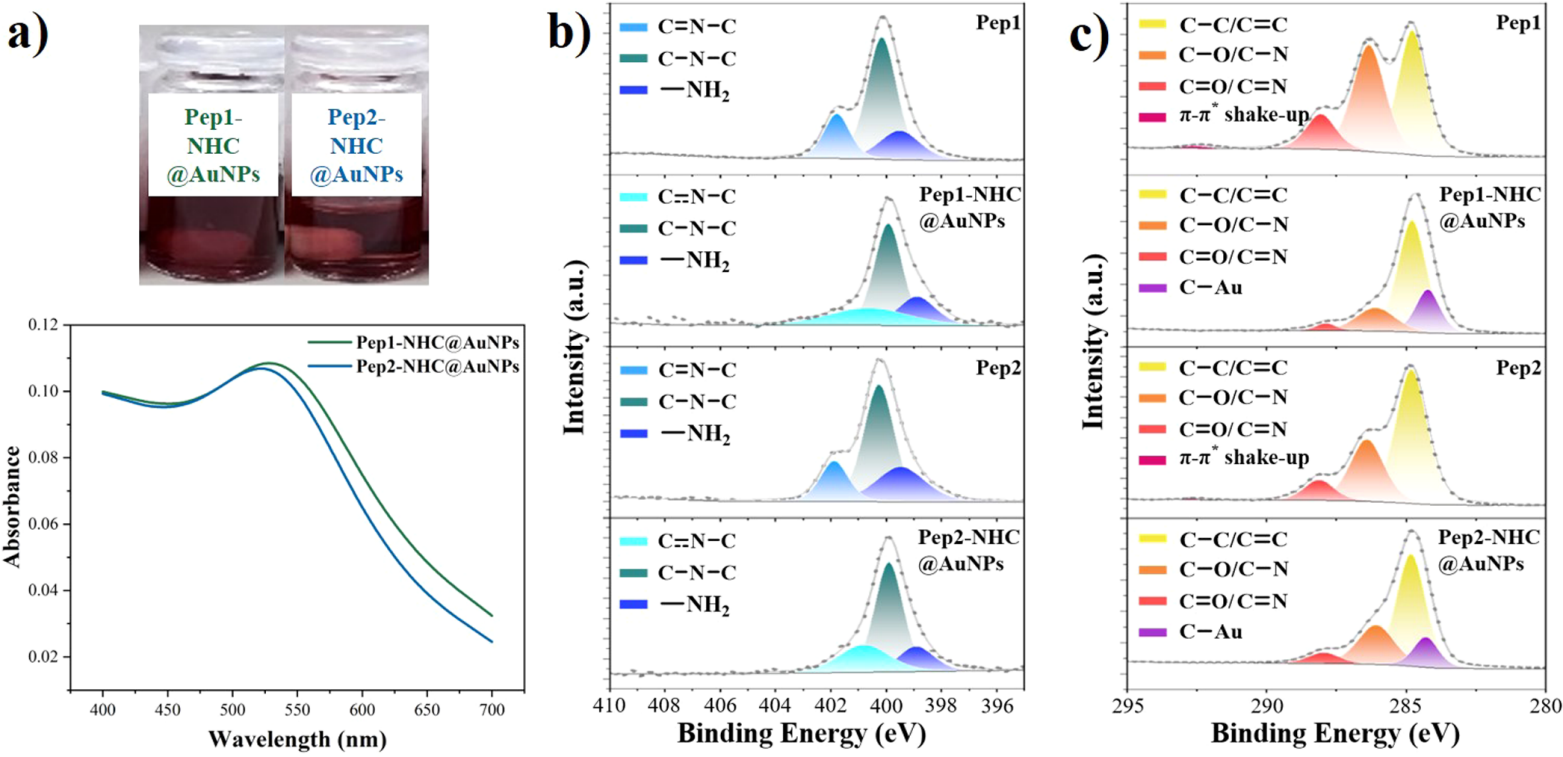
Characterization of peptoid-NHC@AuNPs: (a) UV–vis absorption spectroscopy of peptoid-NHC@AuNPs in toluene. (b) N 1s HR-XPS spectra of **Pep1**, **Pep1-NHC@AuNPs**, **Pep2**, and **Pep2-NHC@AuNPs**. (c) C 1s HR-XPS spectra of **Pep1**, **Pep1-NHC@AuNPs**, **Pep2**, and **Pep2-NHC@AuNPs**.

To investigate the successful NHC formation and binding of the NHC to the AuNP surface, X-ray photoelectron spectroscopy (XPS) analyses were performed on **Pep1**, **Pep1-NHC@AuNPs**, **Pep2**, and **Pep2-NHC@AuNPs**, respectively. In the high-resolution scan of the N 1s region of the free peptoids, binding energies corresponding to the C=N–C moiety on the imidazole ring were observed at 401.78 eV (**Pep1**) and 401.88 eV (**Pep2**) (Figure 1b, Table S1).^40^ However, upon the formation of peptoid-NHC@AuNPs, these peaks shifted to 400.68 eV (**Pep1-NHC@AuNPs**) and 400.81 eV (**Pep2-NHC@AuNPs**) (Figure 1b, Table S1), respectively. These shifts are attributed to the formation of a carbene, which is coordinated to the Au surface, and similar shifts have been reported by others.^15,41,42^ Further, C 1s XPS analysis revealed noticeable peak shifts, and binding energies corresponding to C=N bonds on the imidazole rings were observed at 288.06 eV (**Pep1**) and 288.10 eV (**Pep2**), respectively (Figure 1c, Table S2). Following the formation of peptoid-NHC@AuNPs, C 1s peak shifts to 284.23 eV (**Pep1-NHC@AuNPs**) and 284.30 eV (**Pep2-NHC@AuNPs**) were detected, confirming the successful NHC formation (Figure 1c, Table S2).^43^

The self-assembly behavior of the peptoid-NHC@AuNPs system was further studied via high-resolution transmission electron microscopy (HR-TEM). Both **Pep1-NHC@AuNPs** and **Pep2-NHC@AuNPs** contain spherical AuNP superstructures (Figure 2a–2c, 2e–2g) with average sphere sizes of 248.1 nm (**Pep1-NHC@AuNPs**) and 107.1 nm (**Pep2-NHC@AuNPs**), respectively (Figures S8, S9); the average sizes of the AuNPs within the assembled peptoid-NHC@AuNPs were determined by TEM to be around 4.7 ± 0.50 nm (**Pep1-NHC@AuNPs**) and 4.3 ± 0.51 nm (**Pep2-NHC@AuNPs**) (Figures S10, S11). Further, the lattice spacing of AuNPs in **Pep1-NHC@AuNPs** and **Pep2-NHC@AuNPs** was measured to be approximately 2.3 Å, indicative of the (111) lattice plane of face-centered-cubic (fcc) packing of gold (Figure 2d, 2h).^44^ Selected area electron diffraction (SAED) further confirmed the superlattice structure of fcc-AuNPs in **Pep1-NHC@AuNPs** and **Pep2-NHC@AuNPs**, with typical diffraction patterns indexed to (111), (200), (220), and (222) Bragg reflections of fcc-AuNPs (Figures S12a, S12b).^45,46^

**Figure 2:**
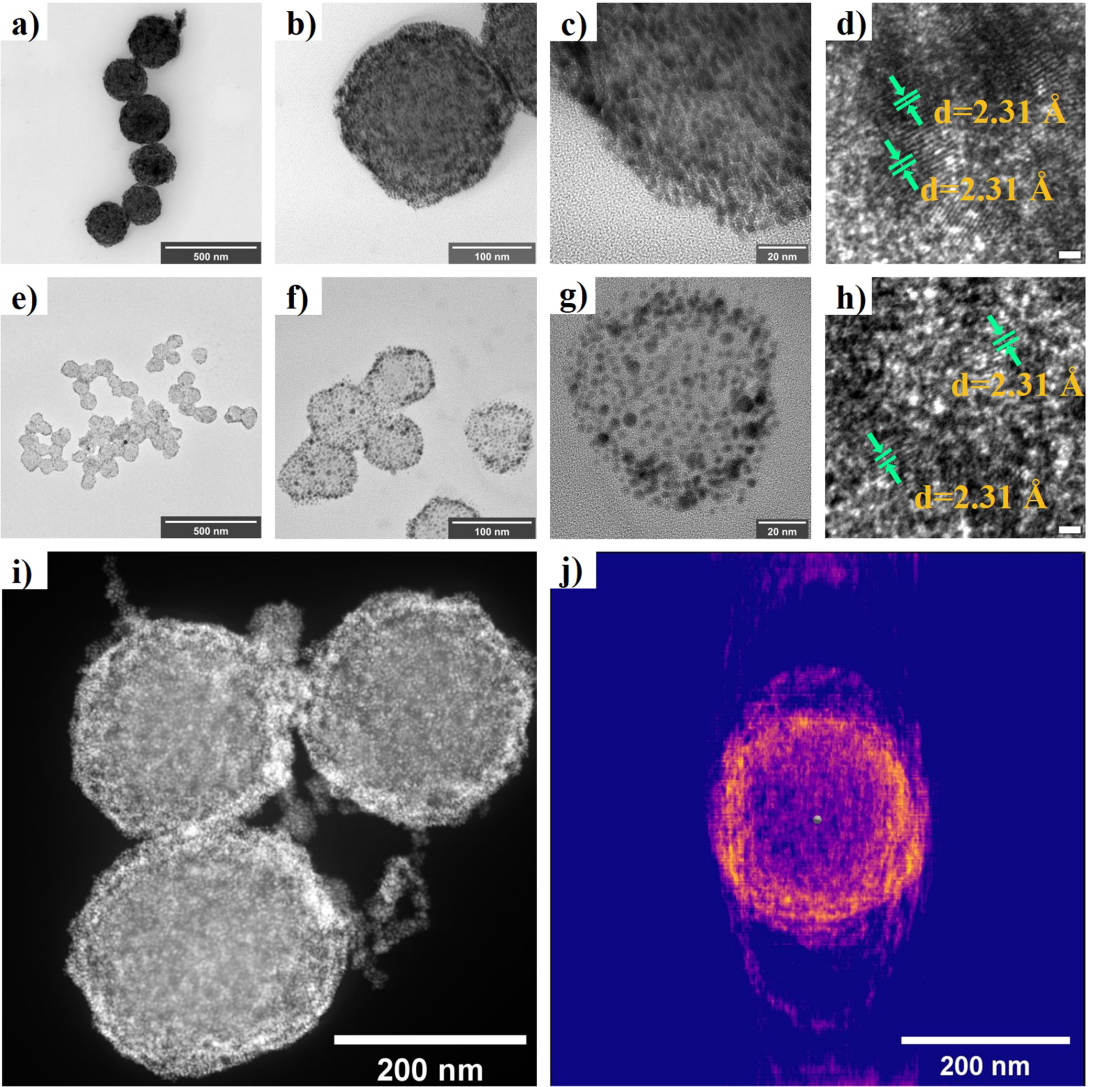
Morphology of peptoid-NHC@AuNPs: (a–d) HR-TEM images of **Pep1-NHC@AuNPs** (scale bar in part d corresponds to 1 nm). (e–h) HR-TEM images of **Pep2-NHC@AuNPs** (scale bar in part h corresponds to 1 nm). (i) HAADF-STEM image of **Pep1-NHC@AuNPs**. (j) 3D structure reconstruction of **Pep1-NHC@AuNPs**.

Interestingly, the HR-TEM images not only revealed the presence of spherical structures but also showed a higher packing density of AuNPs at the spheres’ periphery compared to their central regions. For further insights into the morphology of the peptoid-NHC@AuNPs assembly, high-angle annular dark-field scanning transmission electron microscopy (HAADF-STEM) was employed (Figures 2i, S13a). Consistent with HR-TEM results, HAADF-STEM revealed superstructures of AuNPs which are organized through the peptoid into vesicular-like structures. Additionally, the amorphous core structure at the center of the vesicles most likely arises from the self-assembly of the peptoids (Figures 2i, S13a).^18^ For a comprehensive examination, 3D tomography based on STEM was applied (SI1 for the experimental details). The 3D tomography videos (SI2 for **Pep1-NHC@AuNPs** and SI3 for **Pep2-NHC@AuNPs**) clearly show the peptoid templated supraparticle structure of the peptoid-NHC@AuNPs. Finally, based on the tomography result, 3D structure reconstruction of the peptoid-NHC@AuNPs vesicle was obtained (Figures 2j, S13b).

To gain a better understanding of the self-assembling properties of peptoid-NHC@AuNPs, pure peptoids in acetonitrile, the combination of H­[AuCl_4_] and peptoids as well as the assemblies formed by peptoid-NHC@AuNPs are studied via HR-TEM. Both **Pep1** and **Pep2** exhibited a high propensity to form spherical structures (Figures 3a, 3b); after the addition of H­[AuCl_4_], these spherical structures were retained (Figures 3c, 3d); the final peptoid-NHC@AuNP vesicles formed after the addition of reducing agents (Figures 3e, 3f). Detailed, time-resolved EM monitoring revealed that the self-assembling properties of the peptoid function as a template for the supraparticle assembly of the AuNPs. Further critical micelle concentration (CMC) measurements based on dynamic light scattering (DLS) at 25 °C illustrate the minimum concentration for vesicular assemblies (SI1 for experimental details, Figures S15, S16 for DLS measurement results).^47,48^ CMC concentrations of **Pep1-NHC@AuNPs** and **Pep2-NHC@AuNPs** in toluene were observed at 23.2 and 47.2 μg/mL, respectively (Figure 3g, 3h). Compared to **Pep2-NHC@AuNPs**, **Pep1-NHC@AuNPs** show a smaller CMC value, which we attributed to the side chain difference between **Pep1** (**Npm**) and **Pep2 (Npe**) resulting in a more facile vesicle formation in the case of **Pep1-NHC@AuNPs**.

**Figure 3:**
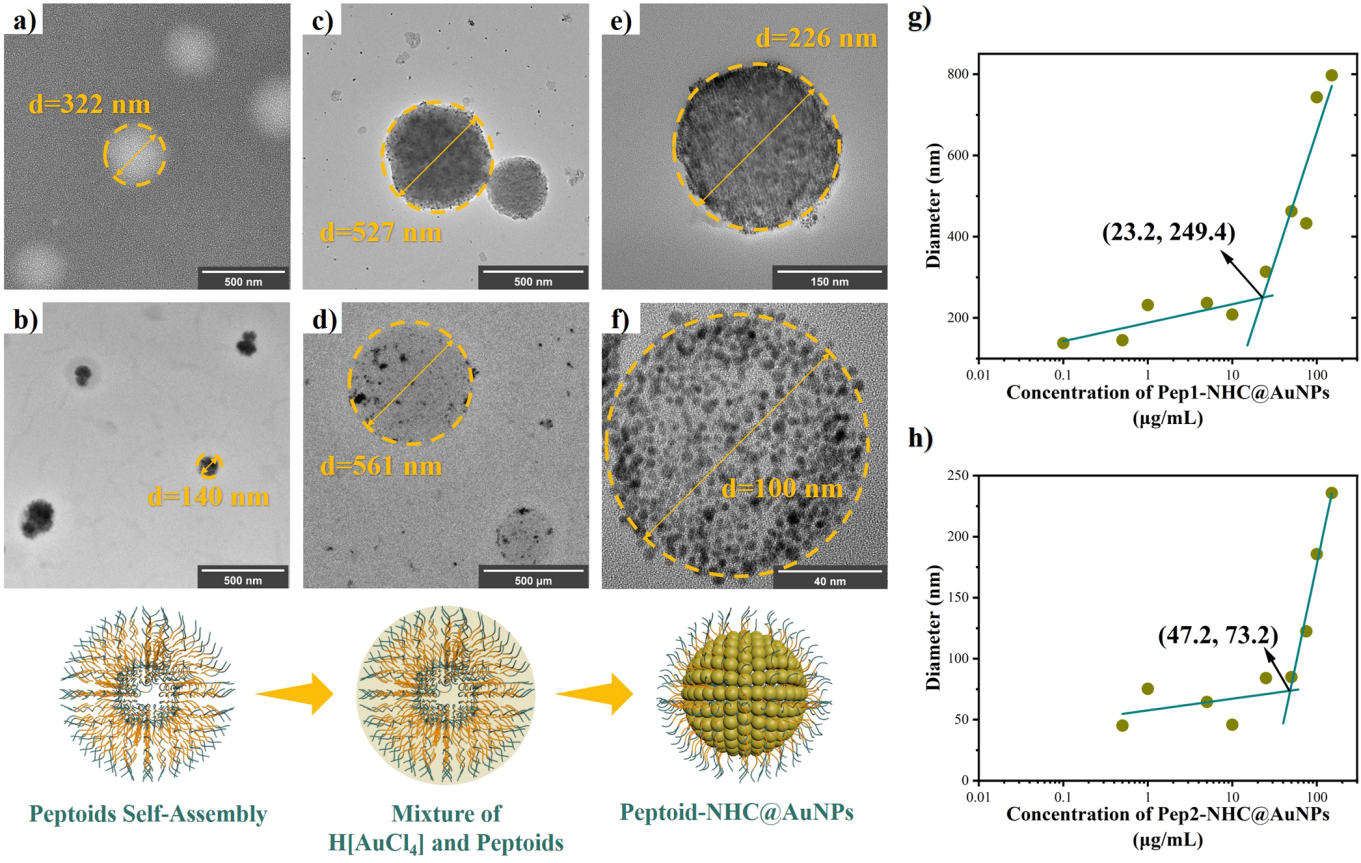
Mechanism study of peptoid-NHC@AuNPs vesicle formation: (a) Negative staining HR-TEM images of **Pep1**. (b) Negative staining HR-TEM images of **Pep2**. (c) HR-TEM images of **Pep1** and the H­[AuCl_4_] mixture. (d) HR-TEM images of the **Pep2** and H­[AuCl_4_] mixture. (e) HR-TEM images of **Pep1-NHC@AuNPs**. (f) HR-TEM images of **Pep2-NHC@AuNPs**. (g) CMC measurement of **Pep1-NHC@AuNPs**. (h) CMC measurement of **Pep2-NHC@AuNPs**.

Hydrophobicity has been identified as a critical influencing factor on the self-assembly behavior of peptoid vesicles.^18,49^ To prove the easy manipulability of the peptoid-NHC@AuNPs vesicles, a series of peptoids are designed with various hydrophobicities based on the structure of **Pep1**. These peptoids, namely, Npm_2_(NomeDig-His)_2_ (**Pep3**) and Npm_6_(NomeDig-His)_2_ (**Pep4**), differ primarily in the number of **Npm** groups present, which give the system higher hydrophobicity with the increase of hydrophobic **Npm** side chain groups (Figure 4a, Chart S2).^50^ Utilizing these peptoids, the successful formation of peptoid-NHC@AuNPs was confirmed using the LSPR peaks, which were observed at 531 nm for **Pep1-NHC@AuNPs**, 527 nm for **Pep3-NHC@AuNPs**, and 535 nm for **Pep4-NHC@AuNPs** (Figure 4b) in toluene, corresponding to AuNPs with sizes of 5.2 ± 0.62 nm, 4.8 ± 0.59 nm, and 5.4 ± 0.55 nm (Figure 4, Figure S17), respectively. Additionally, the formation of peptoid-NHC@AuNPs was confirmed by XPS analysis. In the N 1s spectra, binding energies corresponding to the NHC were observed at 400.68 eV (**Pep1-NHC@AuNPs**), 400.59 eV (**Pep3-NHC@AuNPs**), and 400.28 eV (**Pep4-NHC@AuNPs**), respectively (Table S5, Figure S18), providing evidence for proving the binding of the NHC to the AuNPs. Furthermore, C 1s spectra indicated binding energies corresponding to the carbene formation at 284.25 eV (**Pep1-NHC@AuNPs**), 284.01 eV (**Pep3-NHC@AuNPs**), and 284.06 eV (**Pep4-NHC@AuNPs**), respectively (Table S6, Figure S19). TEM was performed to visualize effects of the peptoid chain length on the self-assembly behavior of peptoid-NHC@AuNPs (Figure 4d–4i). To facilitate size comparisons, size counts were conducted based on 100 peptoid-NHC@AuNPs vesicles for each sample. Hereby it was found that the different numbers of **Npm** groups have a significant influence on the size of the self-assembled vesicles. When only 2 **Npm** groups are present, an average size of 308.3 nm is observed (Figure S20). If the number of **Npm** groups is increased to 4, the vesicle size decreases to 249.5 nm (Figure S21), while the smallest vesicles were observed when 6 **Npm** groups are present at 203.3 nm (**Pep4-NHC@AuNPs**, Figure S22). Similar results could also be obtained via DLS measurements (Figure S23). We attribute size changes to the increased aromatic **Npm** groups present in the peptoids, which can undergo π–π stacking, leading to closer packing of the respective peptoids, as previously observed in the assembly of **Npm**-containing peptoids into crystalline nanomaterials.^50–52^

**Figure 4:**
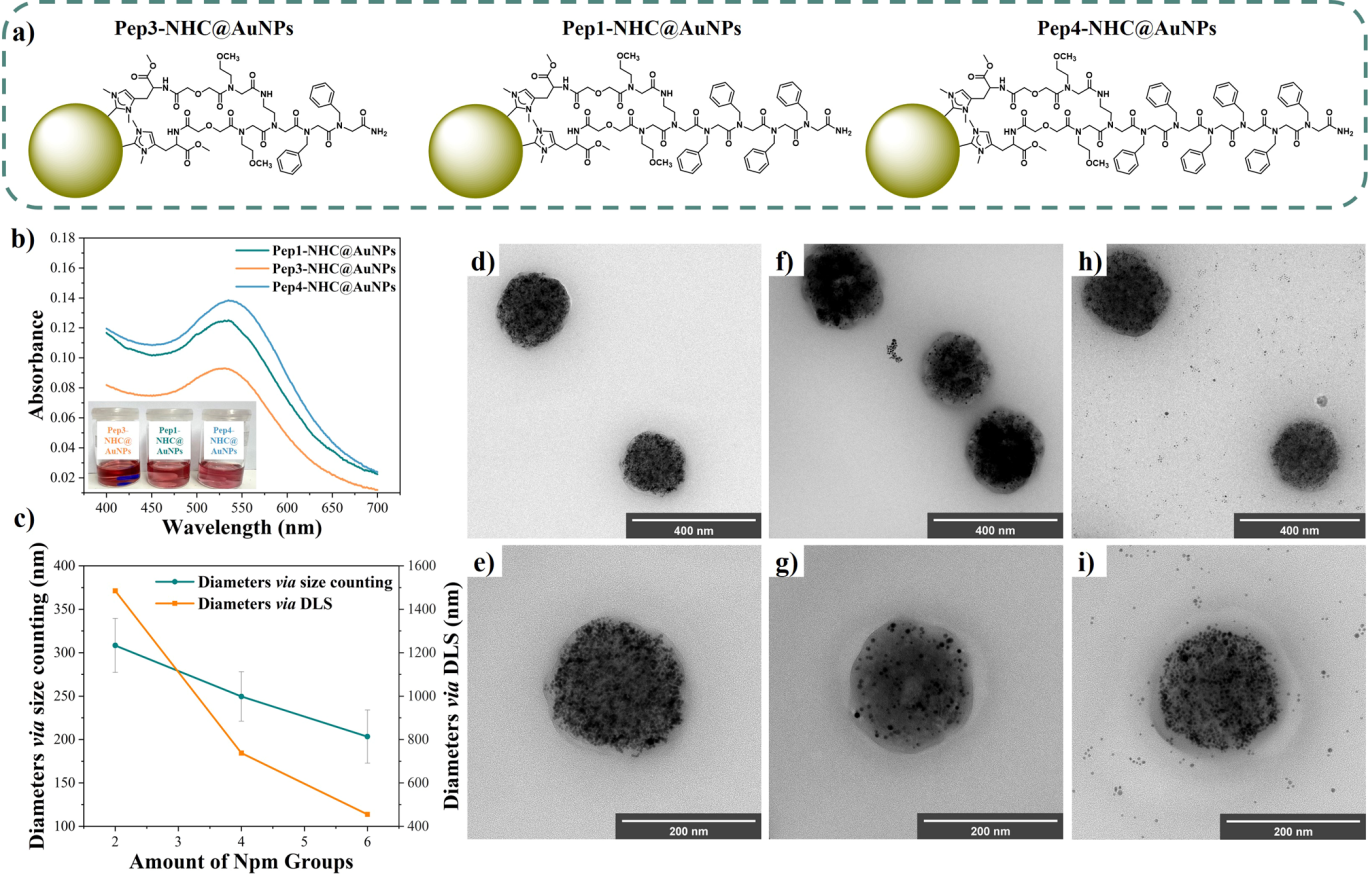
(a) Chemical structure of peptoids-NHC@AuNPs utilized for the size comparison study. (b) UV–vis spectrum of peptoid-NHC@AuNPs in toluene. (c) Size comparison of peptoid-NHC@AuNPs based on size counts via HR-TEM images and DLS analysis. (d, e) HR-TEM images of **Pep3-NHC@AuNPs**. (f, g) HR-TEM images of **Pep1-NHC@AuNPs**. (h, i) HR-TEM images of **Pep4-NHC@AuNPs**.

The peptoid-NHC@AuNPs system also demonstrates structural tunability in response to changes in the solvent environment. Typical LSPR peaks of **Pep4-NHC@AuNPs** in toluene, chloroform, and water were observed at 572, 531, and 542 nm, respectively (Figure S24). TEM measurement showed that in toluene a similar vesicular structure was observed; when **Pep4-NHC@AuNPs** were redispersed in chloroform, the morphology shifted to a cubic-like structure; after phase transfer into water, the peptoid-NHC@AuNPs assembled into a tubular structure (Figure S25). To understand the solvent influence of peptoid-NHC@AuNPs, synchrotron-based X-ray diffraction (XRD) analysis was conducted to investigate the molecular interaction within the **Pep4** assembly. The XRD results confirm that **Pep4** self-assembly exhibits a crystalline structure (Figure S26). The first low-*q* peak, corresponding to a bilayer-like packing arrangement, reveals a spacing of 2.46 nm. This bilayer structure, characteristic of amphiphilic peptoids, resembles the packing seen in previously studied peptoid nanosheets and nanotubes, though with a significantly reduced thickness due to a change in the number of hydrophobic side chains. A peak at *q* = 1.37 Å^–1^ indicates a spacing of 4.55 Å, which corresponds to the orderly alignment of the peptoid backbone chains.^50^ Additionally, the 1.37 nm spacing is attributed to the distance between two peptoid backbones within the bilayer, where the **Npm** groups are positioned facing each other. Two significant peaks at 4.15 and 3.56 Å correspond to the presence of extensive π-stacking among aromatic side-chain groups.^51,52^ Lastly, a spacing of 3.0 Å likely represents the distance between adjacent residues along the backbone in the direction of a cis-conformation peptoid chain. Such kind of molecular interaction results in the solvent-related self-assembly behavior that with the increase of solvent polarity, the driving force of restricting hydrophobic domains leads to the morphology transformation.^53,54^ However, the absence of a hydrophilic domain restricts the phase transfer of **Pep4-NHC@AuNPs** from the organic phase to the aqueous phase.

To further demonstrate the critical role of having an NHC group combined with self-assembling peptoids for the synthesis of this type of peptoid-NHC@AuNP vesicles, we further designed a thiol-containing peptoid, Npm_4_(NomeDigSH)_2_ (**Pep5**, Figure S7), because thiols have been commonly used to attach complex molecules to AuNPs.^55^ Peptoid-S@AuNPs were obtained following the reaction protocol developed for peptoid-NHC@AuNPs with small modifications accounting for the changes of linkage sides (see details in the SI and Chart S3). Successful formation of AuNPs was verified through the characteristic LSPR peak at 526 nm (Figure S27), and the successful formation of the Au–S bond was confirmed by XPS, where a characteristic main S 2p doublet at 162.49 eV indicated successful thiol–gold coordination (Figure S28, Tables S5–S7).^56–58^ The ability of **Pep5-S@AuNPs** to form superstructures was investigated in detail by EM measurements. First, to verify the presence of Au and peptoid, STEM-EDS (energy-dispersive X-ray spectroscopy) mapping, which showed a similar elemental distribution of Au and S (Figure S29), was conducted. Further, TEM and STEM measurements showed that a limited number of irregular vesicular-like structures were formed, and a significant amount of free randomly distributed AuNPs were detected (Figure 5c, 5d, Figure S29). This observation is in strong contrast to that of **Pep1-NHC@AuNPs** vesicles, where the AuNPs are confined within the vesicle structure (Figure 5a–5d). The reduced binding affinity and nonspecificity of thiol with Au­(III/I) and Au(0) disrupted this simultaneous AuNP formation and assembly process, thus leading to the formation of irregular vesicles covered with a low density of AuNPs. To investigate this further, etching experiments with 1-dodecanethiol as a competing surfactant were performed. UV–vis spectra were recorded throughout 24 h, whereby no significant change in the LSPR peak position could be observed for peptoid-NHC@AuNPs (Figure 5e). However, an obvious red shift from 526 to 545 nm was observed for peptoid-S@AuNPs (Figure 5g). This red shift can be attributed to the ripening of AuNPs during the etching experiment and is most likely caused by a dynamic surfactant exchange between **Pep5** and 1-dodecanethiol. To further characterize the changes during the etching experiment, DLS measurements over 24 h were recorded. The peptoid-NHC@AuNPs vesicles remained stable after thiol etching, whereas the vesicular-like structures formed by peptoid-S@AuNPs exhibited a significant size increase (Figures S30–S33). Additionally, TEM measurements of peptoid-NHC@AuNPs and peptoid-S@AuNPs after 24 h of thiol etching confirmed that the hybrid vesicular structure of peptoid-NHC@AuNPs could still be clearly identified (Figures 5f, S34). In contrast, for peptoid-S@AuNPs, those irregular vesicular-like structures nearly disappeared (Figures 5h, S35) and became peptoid assemblies mostly free of AuNPs, as confirmed through STEM-EDX mapping (Figure S36). We attribute the loss of AuNPs to the etching effect of 1-dodecanethiol, where dynamic ligand exchange between **Pep5** and 1-dodecanethiol takes place. In contrast, the stability of the peptoid-NHC@AuNPs system toward thiol etching is due to the robust NHC-type linkage between AuNPs and peptoid, which shows exceptional robustness on Au(111) surfaces (Figure S37, Tables S8, S9).^33,34^

**Figure 5:**
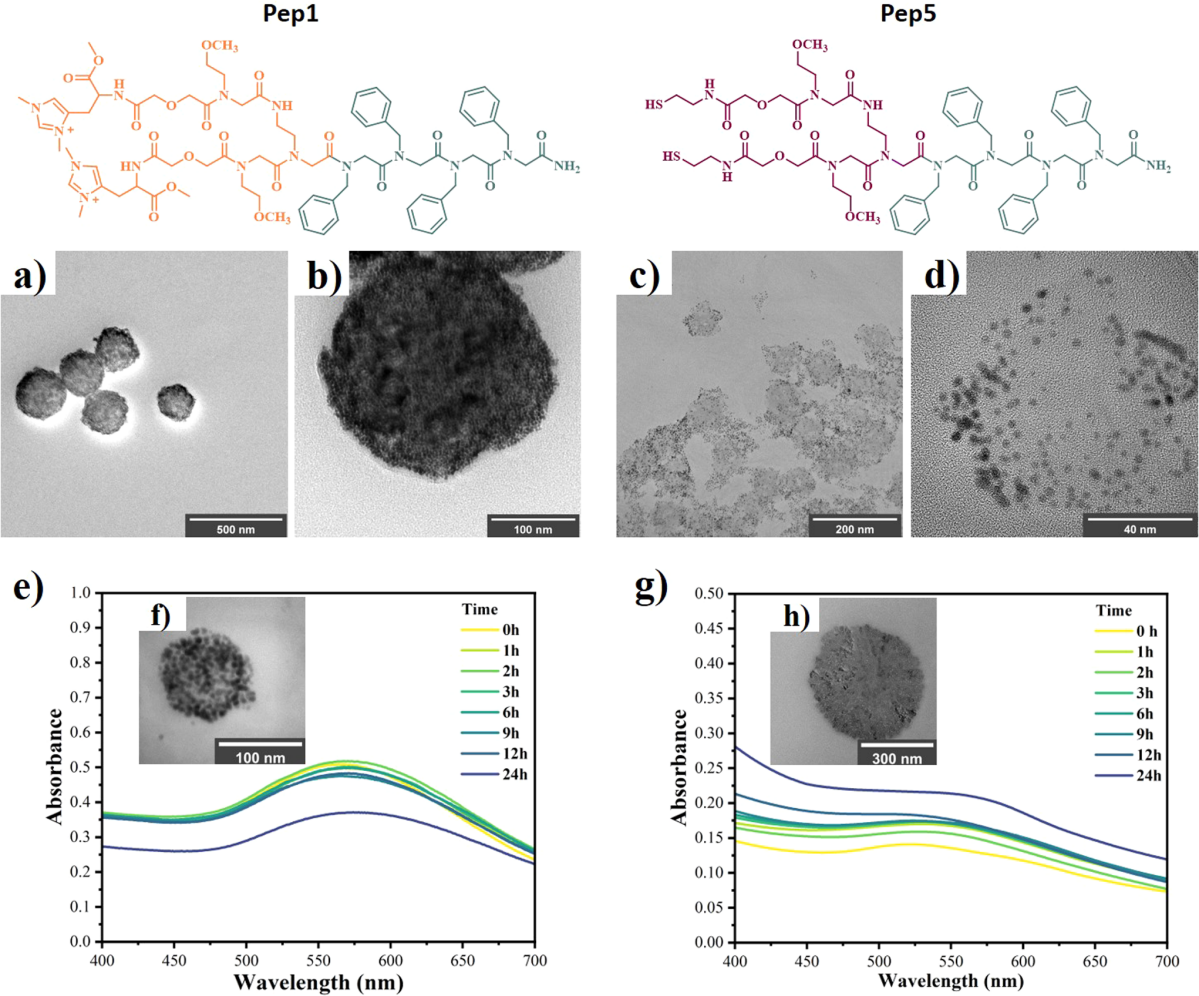
Structure of **Pep1** and **Pep5**. (a) TEM and (b) HRTEM images of **Pep1-NHC@AuNP** vesicles showing densely packed NPs. (c) TEM and (d) HRTEM images of irregular **Pep5-S@AuNP** vesicle-like structures showing a low density of NPs. (e) UV–vis spectroscopy of **Pep1-NHC@AuNPs** mixed with 0.5 mM 1-dodecanethiol at 0 h, 1 h, 2 h, 3 h, 6 h, 9 h, 12 h, and 24 h in toluene. (f) TEM images of **Pep1-NHC@AuNPs** after thiol etching. (g) UV–vis spectroscopy of **Pep5-S@AuNPs** mixed with 0.5 mM 1-dodecanethiol at 0 h, 1 h, 2 h, 3 h, 6 h, 9 h, 12 h, and 24 h in toluene. (h) TEM images of **Pep5-S@AuNPs** after thiol etching.

Finally, we wanted to investigate if the AuNP superstructures can be utilized for surface-enhanced Raman scattering (SERS) hot spot engineering, as nanoparticle arrangements directed by molecular self-assembly have been proven to be an effective strategy for this purpose.^59,60^ To investigate the SERS activity of peptoid-NHC@AuNPs, comparison experiments (SI1 for detailed experimental details and Figure S38 for Raman measurement samples) between peptoids and peptoid-NHC@AuNPs were performed. For **Pep1** and **Pep2**, Raman spectra with low signal intensity were obtained, resulting in spectra that were difficult to analyze (Figure S39a). However, when peptoid-NHC@AuNPs vesicles were measured, Raman spectra displayed distinct enhanced peaks, which correspond to the structures of **Pep1-NHC@AuNPs** and **Pep2-NHC@AuNPs** (Figure S39a). In detail, the peak area from 1000 to 1200 cm^–1^ is contributed by C–O and C–N stretching vibrations; the peak area from 1200 to 1500 cm^–1^ is contributed by CH_2_ and CH_3_ bending vibrations, N–H bending vibrations (Amide III band), and C–C stretching vibrations of the benzene rings, while the peak area from 1500 to 1700 cm^–1^ is contributed by stretching vibrations of C=O (Amide I band) and an unsaturated bond between the center carbene carbon and adjacent N.^61–63^ Furthermore, based on the premise that 1500–1700 cm^–1^ is set as the signal area while 1800–2000 cm^–1^ is set as the noise area, the signal-to-noise ratios (SNRs) of **Pep1**, **Pep2**, **Pep1-NHC@AuNPs**, and **Pep2-NHC@AuNPs** were calculated to be 1.75, 1.80, 57.83, and 29.51, respectively (Table S10), indicating significant amplifications of the Raman signals when AuNPs are present. To more intuitively show the enhancement of the Raman signal, Raman mapping based on the Amide I band was performed (Figure S39b–S39e). For **Pep1** and **Pep2**, the sample signals and the background are indistinguishable, whereas clearly enhanced Raman signal regions are observed for **Pep1-NHC@AuNPs** and **Pep2-NHC@AuNPs**. Further Raman measurements of **Pep5-S@AuNPs** were conducted, showing significantly lower signal intensity compared to that of **Pep1-NHC@AuNPs**, which can be attributed to the loosely packed and randomly distributed assembly of AuNPs in the **Pep5–S@AuNPs** system (Figure S40, Table S11).

In conclusion, we demonstrate a nanohybrid fabrication method based on a strong NHC–metal bond and peptoid self-assembly. In this method, peptoid-NHC@AuNP hybrid nanovesicles were first obtained, with histidine-2-ylidene-functionalized peptoids providing the NHC–Au bond as a linkage and the peptoid self-assembly forming an ordered structure. Detailed investigation into the structural properties revealed the formation of a vesicular structure with AuNPs located on the exterior of the vesicle. Such peptoid-NHC@AuNP exhibits some degree of morphological tunability under environmental control. Although currently this tunability is limited by the absence of a hydrophilic domain, we are extending this research by adding hydrophilic domains into the peptoid sequence to achieve better structural control in biologically relevant media. Further comparative experiments—including studies with thiol-based systems and stability tests against exogenous thiols—proved the advantage of using NHC bonds to construct hybrid systems. In detail, NHC-based peptoid–AuNP hybrid systems exhibited superior self-assembly behavior and enhanced stability against exogenous thiols. Finally, the construction of peptoid-NHC@AuNPs enables the close packing of AuNPs within confined structures, demonstrating that it is an effective tool for hot spot engineering in SERS research. Moving forward, because of a variety of self-assembling peptoid sequences are available,^18,19^ we expect our method can be used to design and synthesize NP superstructures with various morphologies including NP sheets and tubes. On the other side, NHCs are known for their high binding affinity with a variety of metals, including Ag, Cu, Co, and Ni,^64,65^ and we expect our approach can enable the synthesis of NP vesicles with different metallic compositions for applications including SERS.^66,67^

## Supplementary Material







## Notes

Experimental details, spectral data (SI1, PDF)
3D tomography videos of Pep1-NHC@AuNPs (SI2, AVI)
3D tomography videos of Pep2-NHC@AuNPs (SI3, AVI)
