# Supplementary material for: Directed Synthesis of Gold Nanoparticle Superstructures Using Self-Assembling Peptoids Containing Metal-Bonding N‑Heterocyclic Carbenes
Source: Nano Lett. 2025 Jul 11;25(31):12049–58. doi: 10.1021/acs.nanolett.5c02998 (PMC12333405; doi:10.1021/acs.nanolett.5c02998)
Supplement: Supplementary file 1 [file nl5c02998_si_001.pdf]

Support Information

Directed synthesis of gold nanoparticle superstructures using self-assembling peptoids containing metal-bonding N-heterocyclic carbenes

Lingcong Ge, [1,6] *Thi Kim Hoang Trinh,* [2] *Changning Li,*[2,3] *Florian Mayer,*[3] *Jia Min Chin,*[5] *Chun-Long Chen,* [2,3*] *Michael R. Reithofer*[1*]

[1]*Institute of Inorganic Chemistry, Faculty of Chemistry, University of Vienna, Währinger Straße 42, Vienna 1090, Austria, E-mail: michael.reithofer@univie.ac.at*

[2]Physical Sciences Division, Pacific Northwest National Laboratory, Richland, Washington 99352, United States, E-mail: chunlong.chen@pnnl.gov

[3]Department of Chemical Engineering, University of Washington, Seattle, WA 98195, United States

[4]Institute of Materials Chemistry and Research, Faculty of Chemistry, University of Vienna, Währinger Straße 42, Vienna 1090, Austria

[5]Department of Functional Materials and Catalysis, Faculty of Chemistry, University of Vienna, Währinger Straße 42, Vienna 1090, Austria

[6]Vienna Doctoral School in Chemistry (DoSChem), University of Vienna, Währinger Str. 42, 1090 Vienna, Austria

Content

1. Chemical and Material 4
2. Instruments 4
3. Experimental Details 4
4. Characterization 9
5. NMR result of *compound 1* 12
6. MS result of *compound 1* 13
7. Peptoid purification and mass spectrometry analysis 14
8. Synthesis and chemical structure of peptoid-NHC@AuNPs 20
9. Table of binding energy analysis for C 1s and N 1s HR-XPS scanning of Pep1, Pep1-NHC@AuNPs, Pep2, Pep2-NHC@AuNPs. 21
10. Size counts of Pep1-NHC@AuNPs vesicles 22
11. Size counts of Pep2-NHC@AuNPs vesicles 22
12. Size counts of AuNPs in Pep1-NHC@AuNPs vesicles 23
13. Size counts of AuNPs in Pep2-NHC@AuNPs vesicles 23
14. Selected area electron diffraction (SAED) result of Peptoid-NHC@AuNPs vesicles 24
15. HAADF-STEM and 3D structure reconstruction of Pep2-NHC@AuNPs 24
16. Synthesis results of peptoids and AuNPs nanohybrids without NaH 25
17. DLS measurement for CMC calculation of Pep1-NHC@AuNPs 26
18. DLS measurement for CMC calculation of Pep2-NHC@AuNPs 27
19. Size counts of AuNPs in peptoid-NHC@AuNPs vesicles used for size comparison 28
20. N 1s X-ray photoelectron spectra of peptoid-NHC@AuNPs for size comparison 29
21. C 1s X-ray photoelectron spectra of peptoid-NHC@AuNPs for size comparison 30
22. Size counts of Pep1-NHC@AuNPs vesicles for size comparison 31
23. Size counts of Pep3-NHC@AuNPs for size comparison 32
24. Size counts of Pep4-NHC@AuNPs for size comparison 33
25. DLS Analysis of Pep1-NHC@AuNPS, Pep3-NHC@AuNPs, Pep4-NHC@AuNPs for size comparison. 34
26. UV-Vis absorption spectroscopy of Pep4-NHC@AuNPs in toluene, chloroform and water 35
27. TEM images of Pep4-NHC@AuNPs in toluene, chloroform and water 35
28. X-ray diffraction (XRD) analysis of Pep4 assembly 36
29. Synthesis and chemical structure of peptoid-S@AuNPs 37

30. UV-Vis absorption spectroscopy of Pep5-S@AuNPs 37
31. XPS analysis of Pep5-S@AuNPs 38
32. STEM measurement and STEM-EDS Mapping result of Pep5-S@AuNPs 39
33. DLS measurements for thiol etching of Pep1-NHC@AuNPs over 24 h 40
34. DLS measurement for thiol etching of Pep5-S@AuNPs over 24 h 40
35. DLS measurement results for thiol etching of Pep1-NHC@AuNPs over 24 h 41
36. DLS measurement results for thiol etching of Pep5-S@AuNPs over 24 h 42
37. TEM and STEM images of Pep1-NHC@AuNPs after 24-hour thiol etching 43
38. TEM and STEM images of Pep5-S@AuNPs after 24-hour thiol etching 44
39. STEM-EDS measurement of Pep5-S@AuNPs after 24-hour thiol etching 45
40. C 1s and N 1s X-ray photoelectron spectra of Pep1-NHC@AuNPs after thiol etching 46
41. Microscope images of drop-casting result and selected Raman mapping area (within green square) 47
42. Raman measurement results of Pep1, Pep2, Pep1-NHC@AuNPs, and Pep2-NHC@AuNPs. 48
43. Calculated SNR result of Pep1, Pep1-NHC@AuNPs, Pep2, Pep2-NHC@AuNPs. 49
44. Raman Measurement results of Pep5-S@AuNPs 49
45. Calculated SNR result of Pep5-S@AuNPs. 49
46. Reference 50

1. **Chemical and Material**

Potassium carbonate was bought from Alfa Aesar. Histamine dihydrochloride, di-tert-butyl decarbonate, magnesium Sulfate, ion-exchange resin (Dowex® 1X4 chloride from), sodium borohydride, 1-dodecanethiol, and gold(III) chloride trihydrate were obtained from Sigma-Aldrich. Boc-His-OH were acquired from Iris-Biotech. Iodomethane, and triethylamine were bought from Acros-Fisher. Trifluoroacetic acid (peptide grade) was purchased from FluoroChem. All dry solvents were purchased from Acro Fisher or Sigma Aldrich. All solvents mentioned in the reaction procedure were used in chromatographic purity.

All solvents utilized for peptoid synthesis were procured from Fisher or VWR and employed without further purification. Rink Amide resin (0.7-1.0 meq/g), 1-hydroxybenzotriazole hydrate, and bromoacetic acid were acquired from Chem-Impex International, Inc. *N,N′*-diisopropylcarbodiimide (DIC), 4-methylpiperidine (PIP), benzylamine, 2-methoxyethylamine, and trifluoroacetic acid (TFA) were sourced from Oakwood Chemical. Diglycolic anhydride, *N*-ethyl-*N′*-(3-dimethylaminopropyl)carbodiimide hydrochloride, 4-(dimethylamino)pyridine, and dimethyl sulfoxide were obtained from Fisher Scientific. Phenylethylamine and ethylenediamine were purchased from Sigma Aldrich. All primary amine sub-monomers were utilized as received.

2. **Instruments**

All synthesis is carried out under air conditions if no further statement exists. Air and moisture-sensitive reactions were carried out in flame-fried glassware, under an argon atmosphere, using standard Schlenk techniques or an MBraun UniLab Pro glovebox.

3. **Experimental Details**

3.1. Synthesis of (S)-4-(2-amino-3-methoxy-3-oxopropyl)-1,3-dimethyl-1H-imidazol-3-ium chloride(Compound 1)

In a round-bottom flask, Boc-his-OH (2 g, 7.875 mmol, 1 eq) and potassium carbonate (2.166 g, 15.670 mmol, 2 eq) were suspended in acetonitrile and stirred at room temperature. Subsequently, methyl iodine (2.928 mL, 47.010 mmol, 6 eq) was added to the suspended solution, and the resulting mixture was heated to reflux over the weekend. Concurrently, an ion-exchange resin was prepared by placing 100 mg of the resin into 100 mL of 5.5% HCl. After 48 h, the volatile components were removed under reduced pressure. The residue was then suspended in dichloromethane (DCM) and filtered using celite. The filtrate was concentrated and redissolved in a mixture of 100 mL DCM and trifluoroacetic acid (TFA) at a 5% v/v ratio and stirred for 2 h. After removing all volatiles under reduced pressure, a brown oil was obtained. A small amount of methanol was used to dissolve the crude product. The resulting liquid was passed through an ion-exchange resin column for ion-exchange purification. Subsequently, the crude product was obtained under reduced pressure. The final product was obtained by mixing the oil product with diethyl ether, followed by drying under reduced pressure. (Yield = 57.3 %)

M/Z (ESI) Calcd for $C_9H_{16}N_3O_2Cl$ [M-nCl, $C_9H_{16}N_3O_2^+$]: 198.2455; Found: 198.1234

^{1}H NMR (600 MHz, DMSO) δ 9.13 (s, 1H), 8.96 (s, 2H), 7.60 (s, 1H), 4.41 (s, 1H), 3.82 (d, J = 3.8 Hz, 5H), 3.78 (s, 2H).

^{13}C NMR (600 MHz, DMSO) δ 168.47 (8C), 137.25 (1C), 128.60 (5C), 122.98 (4C), 53.11 (7C), 40.43 (9C), 35.68 (3C), 33.45 (2C), 23.44 (6C).

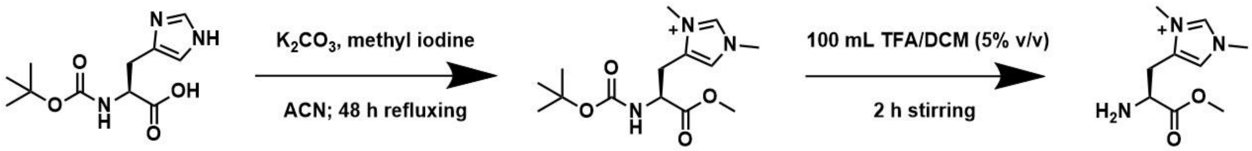

3.2. Synthesis of peptoids

The synthesis of all peptoid sequences was conducted on Rink amide resins (Chem Impex) through solid-phase synthesis, following previously established procedures. Specifically, the Rink amide resins (100 mg, 0.09 mmol) were initially swollen in N,N-dimethylformamide (DMF) for 10 minutes. Subsequently, the Fmoc groups were removed by adding 2 mL of a 20% (v/v) solution of 4-methylpiperidine/DMF, and the mixture was shaken at room temperature for 40 minutes. Following this, the resins were drained and subjected to five washes of 1 mL each with DMF.

Next, the deprotected resins underwent an acylation reaction utilizing 1.5 mL of 0.6 M bromoacetic acid and 0.3 mL of a 50/50 (v/v) mixture of N,N-diisopropylcarbodiimide (DIC)/DMF. The reaction mixture was shaken for 10 minutes at room temperature, followed by washing with DMF (5 × 1 mL). Nucleophilic displacement of bromide with the submonomers was achieved by adding 1.5 mL of a 0.6 M primary amine solution in N-methyl-2-pyrrolidone (NMP) and agitating for 10 minutes at room temperature. The solution was then filtered, and the resins were washed with DMF (5 × 1 mL). The acylation and displacement reactions with appropriate primary amines such as N-(phenylmethyl)glycine (Npm), N-(2-phenylethyl)glycine (Npe), ethylenediamine, and N-(2-methoxyethyl)glycine (Nome) were repeated until the desired target peptoids, including $Npm_2(NomeDig)_2$, $Npm_4(NomeDig)_2$, $Npm_6(NomeDig)_2$, or $Npe_4(Nome)_2$ were achieved.

Next, diglycolic anhydride (208 mg, 1.8 mmol) and 2 mL of DIC/DMF (50/50 v/v) were added separately to the peptoids. Reactions proceeded at room temperature overnight. Subsequently, the solution was filtered, and the resins were washed with DMF (5 × 1 mL). Following this, $Npm_2(NomeDig)_2$, $Npm_4(NomeDig)_2$, $Npm_6(NomeDig)_2$, or $Npe_4(Nome)_2$ were obtained.

In the final step, to obtain the target peptoids, N-ethyl-N'-(3-dimethylaminopropyl)carbodiimide hydrochloride (207 mg, 1.08 mmol), 1-hydroxybenzotriazole hydrate (146 mg, 1.08 mmol), 4-

(dimethylamino)pyridine (44 mg, 0.36 mmol), (S)-4-(2-amino-3-methoxy-3-oxopropyl)-1,3-dimethyl-1H-imidazol-3-ium chloride (107 mg, 0.54 mmol), 2 mL of DMF, and 400 µL of dimethyl sulfoxide were added to the peptoids. Reactions were allowed to proceed at room temperature overnight. The resulting solution was filtered, and the resins were washed with water and DMF (5 × 1 mL). Following this step, $Npm_2(NomeDigHis)_2$ (Pep3), $Npm_4(NomeDig)_2$ (Pep1), $Npm_6(NomeDig)_2$ (Pep4), or $Npe_4(Nome)_2$ (Pep2) were obtained.

To synthesize $Npm_4(NomeDigSH)_2$ (Pep-5), N-ethyl-N'-(3-dimethylaminopropyl)carbodiimide hydrochloride (207 mg, 1.08 mmol), 1-hydroxybenzotriazole hydrate (146 mg, 1.08 mmol), 4-(dimethylamino)pyridine (44 mg, 0.36 mmol), 2-(tritylthio)ethanamine (172 mg, 0.54 mmol) and 2 mL of DMF were added to the $Npm_4(NomeDig)_2$. Reactions were allowed to proceed at room temperature overnight. The resulting solution was filtered, and the resins were washed with water and DMF (5 × 1 mL)

All peptoids underwent purification and were subjected to mass spectrometry analysis, confirming the formation of the target peptoids (SI1, Figure S3 – S7).

3.3. Synthesis of Peptoid-NHC@AuNPs

To synthesize Pep1-NHC@AuNPs, a solvent mixture consisting of 700 µL of acetonitrile and 1000 µL of toluene was prepared. Subsequently, 100 µL of a Pep1 solution (with a concentration of 4.6×10^{-3} mol/L in acetonitrile, 1 eq) and 100 µL of a NaH solution (with a concentration of 13.8×10^{-3} mol/L in acetonitrile, 3 equivalents) were introduced into the mixture, and the mixture was stirred for a minimum of 2 h. Subsequently, 100 µL of an $H[AuCl_4]$ solution (with a concentration of 9.2×10^{-3} mol/L in acetonitrile, 2 eq) was combined with the peptoid solution, subjecting the resulting blend to an additional 30 minutes of stirring before allowing it to settle in a refrigerator (-37 °C) for 4 h. Lastly, 100 µL of a $NaBH_4$ solution was injected (with a concentration of 13.8×10^{-3} mol/L in acetonitrile, 3 equivalents) into the mixture while stirring at 1000 rpm. This led to an immediate color change from yellow to red, showing the successful formation of AuNPs. The final product was precipitated with acetone centrifugation at 12500 rpm, and redispersed in toluene.

Pep2-NHC@AuNPs were synthesized using a similar approach. A mixture of 700 µL acetonitrile and 1000 µL toluene was prepared in the beginning. Next, 100 µL of Pep2 solution (with a concentration of 2.97×10^{-3} mol/L in acetonitrile, 1 eq) and 100 µL of NaH solution (with a concentration of 8.91×10^{-3} mol/L in acetonitrile, 3 equivalents) were to the mixture. After 2 h of stirring, an extra 100 µL of $H[AuCl_4]$ solution (with a concentration of 5.94×10^{-3} mol/L in acetonitrile, 2 eq) was mixed with the peptoid solution and stirred for an additional 30 minutes before refrigerating for 4 h. Finally, rapidly injection 100 µL of $NaBH_4$ solution (with a concentration of 8.91×10^{-3} mol/L in acetonitrile, 3 equivalents) into the mixture was carried out

while stirring at 1000 rpm, resulting in an instant color change from yellow to red as well, indicating successful AuNP formation. The final product underwent purification through acetone centrifugation at 12500 rpm, and was redispersed in toluene, ensuring its readiness for subsequent analysis.

3.4. Thiol-etching experiments of Pep1-NHC@AuNPs

In detail, a mixture of Pep1-NHC@AuNPs (200 μg/mL) and 1-dodecanethiol (0.5 mM) in toluene was prepared for UV-Vis and DLS measurements at 25 ℃. UV-Vis and DLS measurements were carried out at 0 h, 1 h, 2 h, 3 h, 6 h, 9 h, 12 h, and 24 h for stability assessment.

3.5. Controlled experiments for size comparison

Pep1, Pep3, and Pep4 have been specifically designed for controlled experiments aimed at comparing the sizes of vesicles self-assembled with different peptoids. The synthesis process is executed in a simultaneous manner to facilitate meaningful comparisons, maintaining identical conditions except for the variations in ligand types. In detail, 100 μL of 1×10^{-3} mol/L Pep1, Pep3, or Pep4 (with a concentration of 1×10^{-3} mol/L in acetonitrile, 1 eq) and 100 μL of NaH solution (with a concentration of 3×10^{-3} mol/L in acetonitrile, 3 equivalents) were added to a mixture of 700 μL acetonitrile and 1000 μL toluene. After 2 h of stirring, 100 μL of $H[AuCl_4]$ solution (2×10^{-3} mol/L in acetonitrile, 2 eq) was mixed with the peptoid solution and stirred for an additional 30 minutes before refrigerating at -37 ℃ for 4 h. Finally, 100 μL of $NaBH_4$ solution (with a concentration of 3×10^{-3} mol/L in acetonitrile, 3 equivalents) was rapidly injected into the mixture while stirring at 1000 rpm, resulting in an instant color change from yellow to red as well, indicating successful AuNPs formation. The final product underwent purification through acetone centrifugation at 12500 rpm, and was redispersed in 2 mL toluene for subsequent analysis.

3.6. Experiments for morphology control of Pep4-NHC@AuNPs

Pep4-NHC@AuNPs were synthesized using the same approach mentioned in the size comparison section, but Pep4-NHC@AuNPs the final product was initially dispersed in 3 mL of toluene. Next, 2 mL of the colloidal solution was cryo-dried and redispersed in 2 mL of chloroform. For phase transfer to water, 1 mL of Pep4-NHC@AuNPs in chloroform was added dropwise to 2 mL Milli-Q water under vigorous stirring at 1000 rpm at 25 ℃. After 4 h of stirring and 12 h of settling, the chloroform phase was removed. The rest was used for TEM characterization.

3.7. Synthesis of Peptoid-S@AuNPs

Pep5-S@AuNPs were synthesized using a similar approach for reasonable comparison. In detail, 100 μL of 1×10^{-3} mol/L Pep5 (with a concentration of 1×10^{-3} mol/L in acetonitrile, 1 eq) was added to a mixture of 800 μL acetonitrile and 1000 μL toluene. After 2 h of stirring, 100 μL of $H[AuCl_4]$ solution (2×10^{-3} mol/L in acetonitrile, 2 eq) was mixed with the peptoid solution and stirred for an additional 30 minutes before refrigerating at -37 ℃ for 4 h. Finally, 100 μL of $NaBH_4$ solution

(with a concentration of 3×10^{-3} mol/L in acetonitrile, 3 equivalents) was rapidly injected into the mixture while stirring at 1000 rpm, resulting in an instant color change from yellow to red, indicating successful AuNPs formation. The final product was purified by centrifugation with acetone at 12500 rpm, and was redispersed in 2 mL toluene for subsequent analysis.

4. **Characterization**

MS measurements were performed on Bruker Maxis UHR-TOF (high resolution) spectrometer by Mass Spectrometry Centre (MSC) at the Faculty of Chemistry of the University of Vienna.

^{1}H, ^{13}C- and 2D-NMR spectra were recorded at 25 ℃ on a Brucker BioSpin AV Ⅲ 600 or Brucker Biospin AV Neo 500 spectrometer at 600.25/500.32 MHz(1H) and 150.93/125.81 MHz(13C) by NMR centre at the Faculty of Chemistry of the University of Vienna. Residual protic solvent peak ($CDCl_3$, δ^{1}H=7.26, δ^{13}C=77.16; DMSO-d_6, δ^{1}H=2.50, δ^{13}C=39.52) were used as internal standard. Chemical shifts are given in ppm (δ), and coupling constants (J) are given in Hertz (Hz). MestReNova 14.1 was used for NMR spectra analysis and visualization.

A Waters Breeze 2 preparative HPLC, a Waters SQD2 UPLC-MS, a Biotage® V-10 Touch Evaporator, and Labconco FreeZone Plus 4.5 Liter Benchtop Cascade (-84 °C) Freeze Dry System were used for purification and characterization of the peptoids.

The surface composition of peptoid-NHC@AuNPs was characterized by X-ray photoelectron spectroscopy (XPS, Nexsa XPS system, Thermo-Fisher) using a radiation source gun-type Al Kα with integrated flood gun and a pass energy of 200 eV, a spot size of 400 µm, an energy step size. 1 eV for the survey spectrum. Analysis was performed from high-resolution spectra before cleaning the surface with Ar-clusters (1000 atoms, 6000 eV, 1 mm spot size) for 60 s. The high-resolution spectra were acquired with 30 passes at pass energy of 50 eV, energy step size 0.1 eV, a spot size of 400 µm, using Thermo Avantage v5.9914, Build 06617 with Smart background. Samples were prepared on pre-cleaned silicon wafer pieces (~0.25 cm^2) by drop casting of sample dispersion. In the case of the etching experiment, samples were centrifuged, washed with acetone once, and redispersed in toluene before drop-casting the solution onto a silicon wafer.

High-Resolution Transmission Electron Microscopy (HR-TEM) and High-Angle Annular Dark-Field Scanning Transmission Electron Microscopy (HAADF-STEM) measurements were carried out at the Electron Microscopy Facility at IST Austria using a S/TEM Jeol JEM2800 with the accelerating voltage of 200 kV equipped with a CMOS TEM camera TemCam-XF416, and EDS result was gathered by an EDS detector Jeol Centurio, which is a large solid angle silicon drift detector with 100 mm^2 active area for ultrafast elemental mapping of S/TEM samples. All samples were prepared by drop-casting onto 200-mesh copper grids coated with a carbon film (Rigorous). Subsequently, the samples were dried in an oven at 70 °C before usage. All images were adjusted for brightness, contrast, and picture size using ImageJ. The reported size distribution and lattice distance were measured using the EM Measure software package. Reported sizes are averaged over a total of 100 counts. For 3D tomography based on STEM, samples were loaded onto a half-mesh grid for tilting, and images were captured using the Recorder (JEOL) software. Tilting angles ranging from 0° to ±75° were applied for Pep1-NHC@AuNPs, with 4° intervals starting from 0°.

Similarly, -68° to +72° tilting angles were applied for Pep2-NHC@AuNPs, with 4° intervals starting from 0°. In the end. 3d structure reconstruction of Pep1-NHC@AuNPs and Pep2-NHC@AuNPs were carried out by TOMVIZ. Additionally, selected area electron diffraction (SAED) was also performed by JEM2800, with a selected area aperture diameter of 50 nm, and camera length of 10m. The determination of the diffraction spots was carries out by calculated with standard Si(110) sample under the same camera length, with usage of Bragg's equation: $\lambda L = r \cdot d$: firstly, λL of the JEM2800 was calculated with the standard Si(110); second, R is measured by EM Measure software; finally, the d-space was calculated by Bragg's equation.[1]

Transmission electron microscopy (TEM) was measured at the Electron Microscopy Facility at IST Austria using a Phillips Tecnai 12 (120kV) TEM equipped with a CMOS TVIPS TemCam-F216 camera. Prior to measurement, samples suspended in toluene were drop-casted onto formvar carbon film on 200 mesh copper grids.

UV-Vis adsorption measurements of freshly prepared peptoid-NHC@AuNPs redispersed in toluene, with concentrations ranging from 0.05 to 0.1 mg/mL, were carried out on Agilent Cary 60 Spectrophotometer at 25 ℃. For thiol etching experiments, a solution of Pep1-NHC@AuNPs (200 μg/mL) and 1-dodecanethiol (0.5 mM) was prepared for 24 h measurement.

DLS measurements were performed on Zetasizer Nano particle characterization system. The experiment was carried out at 25 ℃ by dropping 1 mL samples into quartz cuvettes. For critical micelle concentration (CMC) measurement, 0.1, 0.5, 1, 5, 10, 25, 50, 75, 100, 150 μg/mL Pep1-NHC@AuNPs, and 0.5, 1, 5, 10, 25, 50, 75, 100, 150 μg/mL Pep2-NHC@AuNPs were prepared for DLS measurement. For thiol etching experiments, a solution of peptoi-AuNPs (200 μg/mL) and 1-dodecanethiol (0.5 mM) was prepared for measurement. For size comparison, parallelly synthesized peptoid-NHC@AuNPs were dispersed in 2 mL toluene for DLS measurements.

X-ray diffraction (XRD) measurements were conducted in accordance with established protocols from previous reports.[2] Powder XRD data were obtained at the Advanced Light Source on beamline 8.3.1, which is designed for multiple wavelength anomalous diffraction and monochromatic macromolecular crystallography. The beamline utilizes a 5 T single-pole superbend magnet, covering an energy range of 5–17 keV. Data collection was carried out using an ADSC Q315r detector arranged in a 3 × 3 CCD array, operating at a wavelength of 1.11583 Å. The detector was placed 400 mm away from the sample. For sample preparation, peptoid assemblies were mounted on a Kapton mesh (MiTeGen) and air-dried before XRD measurements. The resulting data were analyzed using customized Python scripts.

Raman spectroscopy and Raman mapping measurements were carried out by a confocal Raman microscope (alpha 300 RS; WITec Wissenschaftliche Instrumente und Technologie GmbH, Germany), with a 633 nm laser in power of 15 mW. For sample preparation, around 80 μg of

Pep1, and Pep2, Pep1-NHC@AuNPs, and Pep2-AuNPs were drop-casted on 22*22 mm capping glass for further measurement. For Raman spectroscopy measurement, the aquation time of every scan was 20 seconds and every spectrum was obtained with 20 scans. Further data analysis was performed by the WITec Project FIVE software with the same protocol: first move the cosmic ray by CRR with a filter size of 2 and dynamic factor of 4; then subtract the background by polynomial with ORDER 5. For the calculation of signal-to-noise ratio (SNR),[3] the root mean square (RMS) of selected noise and signal were calculated first:

$$RMS = \sqrt{\frac{1}{N}\sum_{i=1}^{N} x^2}$$

X = Raman intensity of selected spots, N = Amount of selected spots

The SNR was calculated:

$$SNR = 20 * log_{10}(\frac{RMS\ of\ Signal}{\mathrm{R}MS\ of\ Noise})$$

For Raman mapping, an area of 25*25 µm was selected. For every mapping, 25 lines containing 25 spots were set for Raman mapping, with an integration of 10 seconds. Finally, the data was analyzed with the same protocol mentioned before. The Raman mapping was generated with the configuration of the Amide I band.

5. **NMR result of *compound 1***

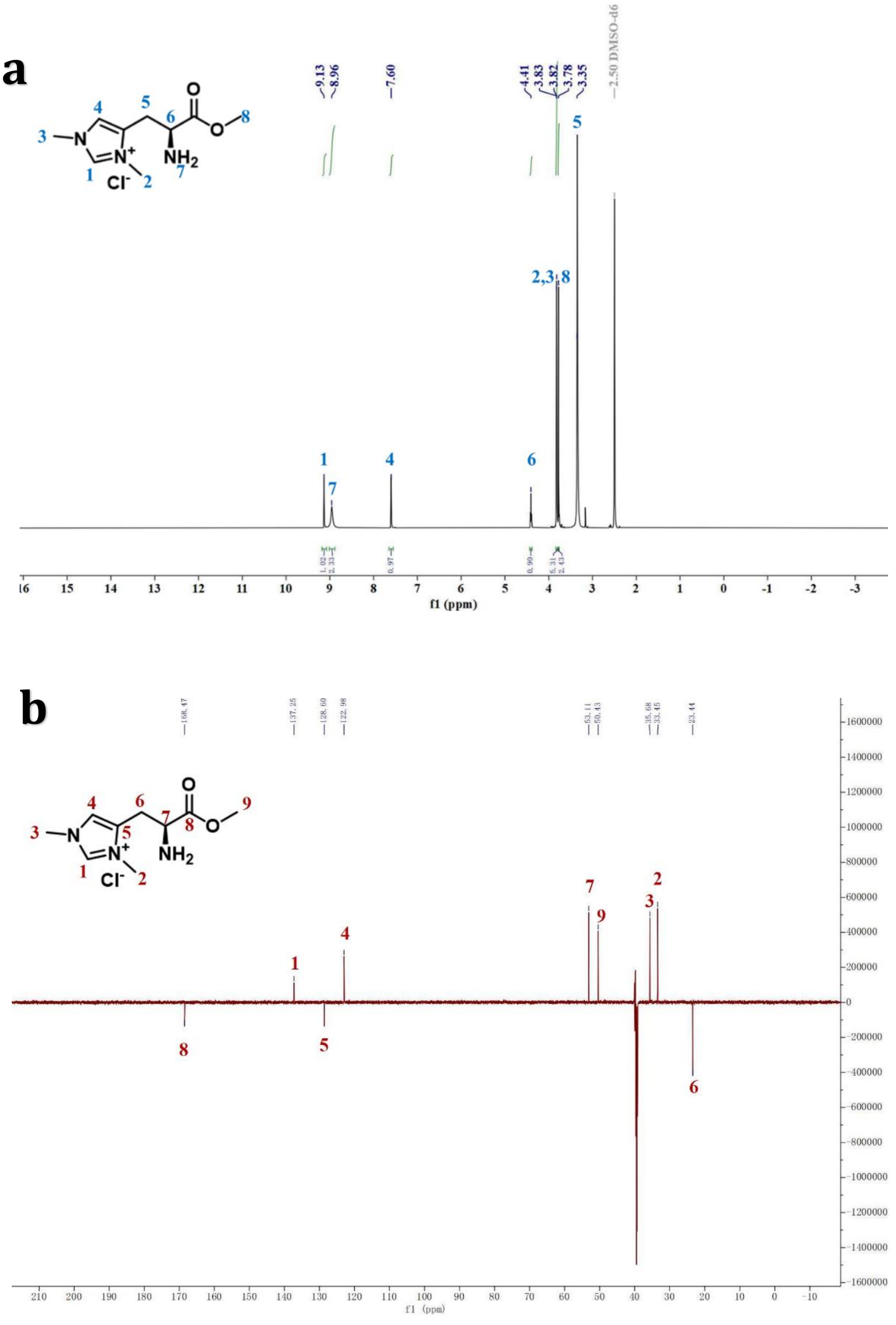

Figure S1. (a). ^{1}H NMR Analysis of ***compound 1*** (b).^{13}C NMR Analysis of ***compound 1***

6. **MS result of *compound 1***

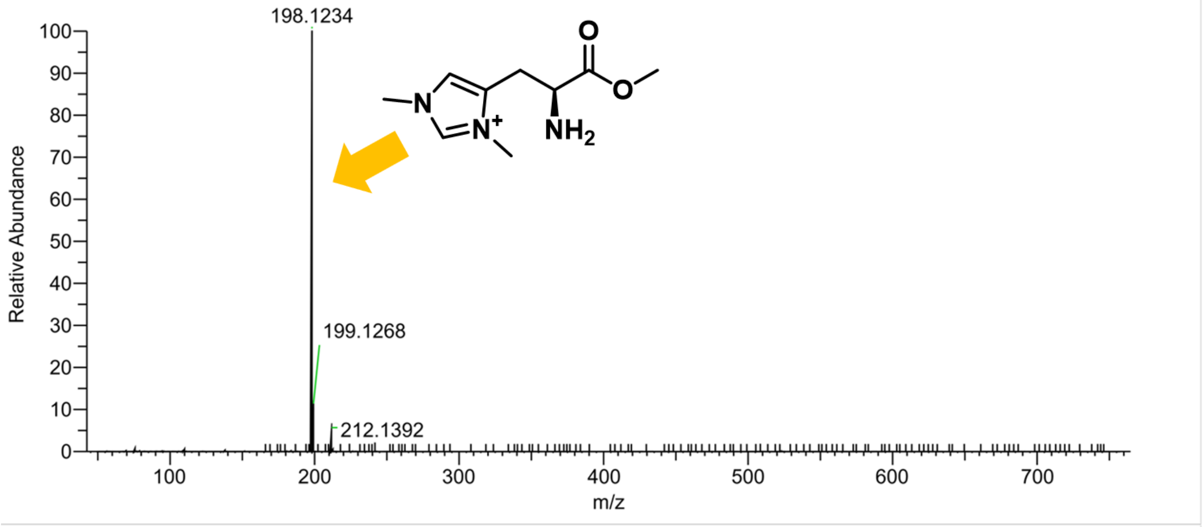

Figure S2. MS Result of ***compound 1***

7. Peptoid purification and mass spectrometry analysis

All peptoids (Pep1 – Pep5) underwent high-performance liquid chromatography (HPLC) purification and were subjected to mass spectrometry analysis, confirming the formation of the target peptoids (**Figure S3 – S7**). Specifically, peptoids were detached from the resin beads by treating them with 3 mL of a 95/5 (v/v) trifluoroacetic acid (TFA)/H_2O (Pep1 to Pep4) or 90/5/5 (v/v) TFA/H2O/triisopropyl silane (Pep5) solution solution for 30 minutes with agitation. Subsequently, the solution was collected and the TFA solvent was evaporated under reduced pressure at 36°C. The resulting crude peptoid products were dissolved in 80/20 (v/v) acetonitrile/H_2O and subjected to purification using reverse-phase HPLC. This was carried out on a Water 1525 system fitted with an XBridge^{TM} Prep C18 OBDTM column (10 µm, 19 mm × 100 mm), employing a linear gradient of 20 – 40 % acetonitrile in water with 0.1% trifluoroacetic acid for Pep3, or 30 – 50% acetonitrile for peptoids Pep1, Pep2 and Pep4. Following purification, waters ultra-performance liquid chromatography-mass spectrometry (UPLC-MS) was used to characterize the purified peptoids according to established protocols outlined in our previous work. [2, 4, 3]

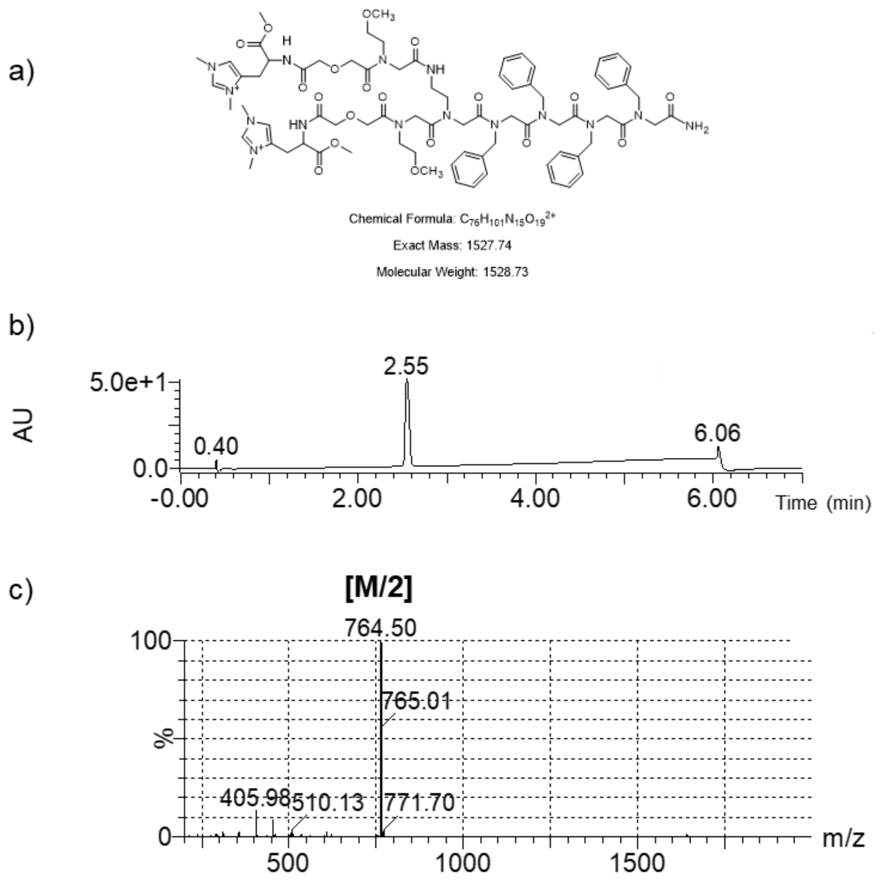

Figure S3. UPLC analysis of Pep1. a) Chemical structure. b) LC-MS chromatogram with the gradient of 5 - 95% CH_3CN in H_2O. c) ESI^+ ionization pattern.

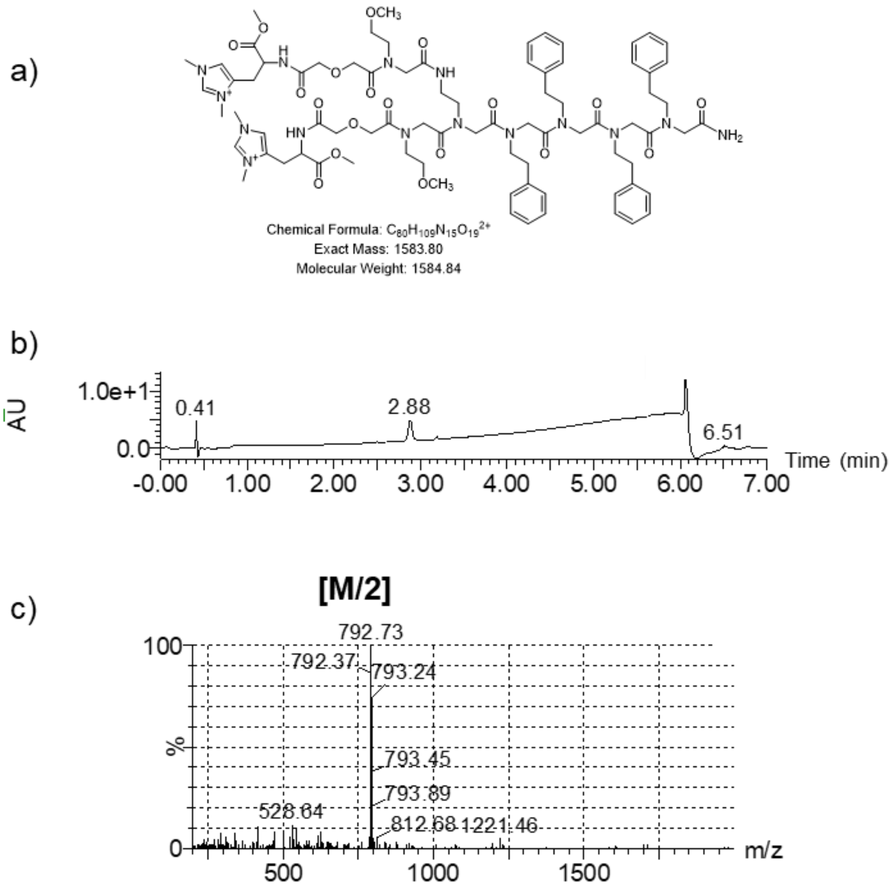

Figure S4. UPLC analysis of Pep2. a) Chemical structure. b) LC-MS chromatogram with the gradient of 5 - 95% CH_3CN in H_2O. c) ESI^+ ionization pattern.

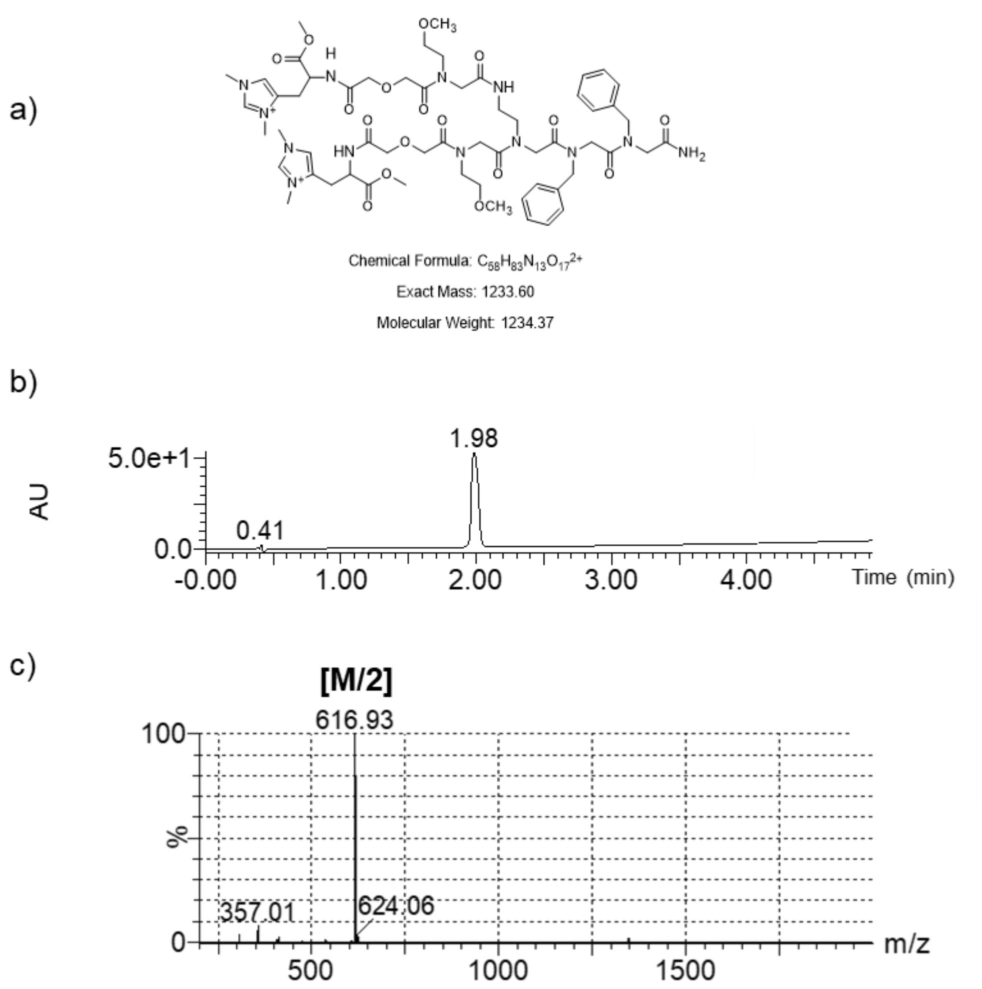

Figure S5. UPLC analysis of Pep3. a) Chemical structure. b) LC-MS chromatogram with the gradient of 5 - 95% CH_3CN in H_2O. c) ESI^+ ionization pattern.

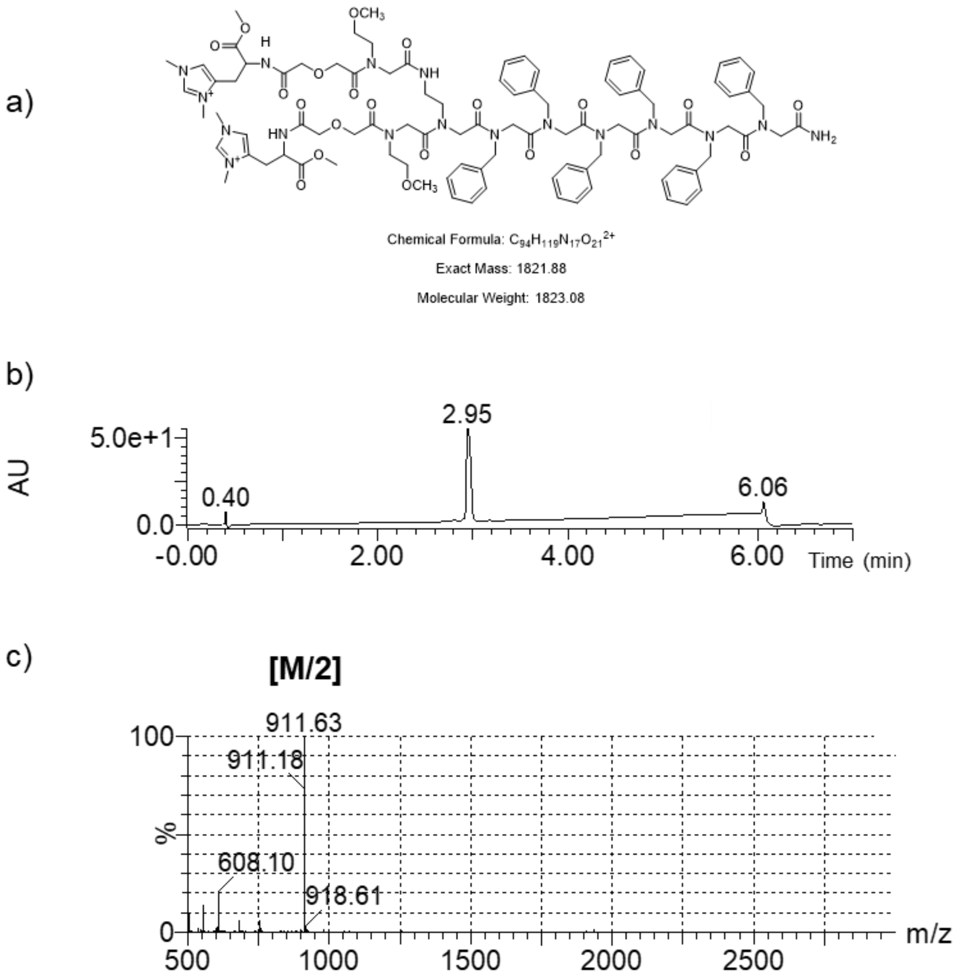

Figure S6. UPLC analysis of Pep4. a) Chemical structure. b) LC-MS chromatogram with the gradient of 5 - 95% CH_3CN in H_2O. c) ESI^+ ionization pattern.

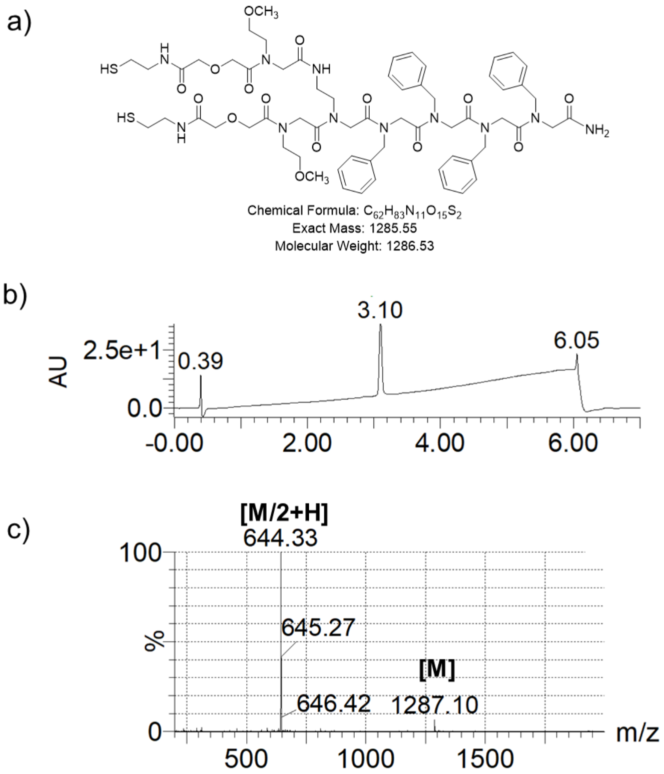

Figure S7. UPLC analysis of Pep5. a) Chemical structure. b) LC-MS chromatogram with the gradient of 5 - 95% CH_3CN in H_2O. c) ESI^+ ionization pattern.

8. Synthesis and chemical structure of peptoid-NHC@AuNPs

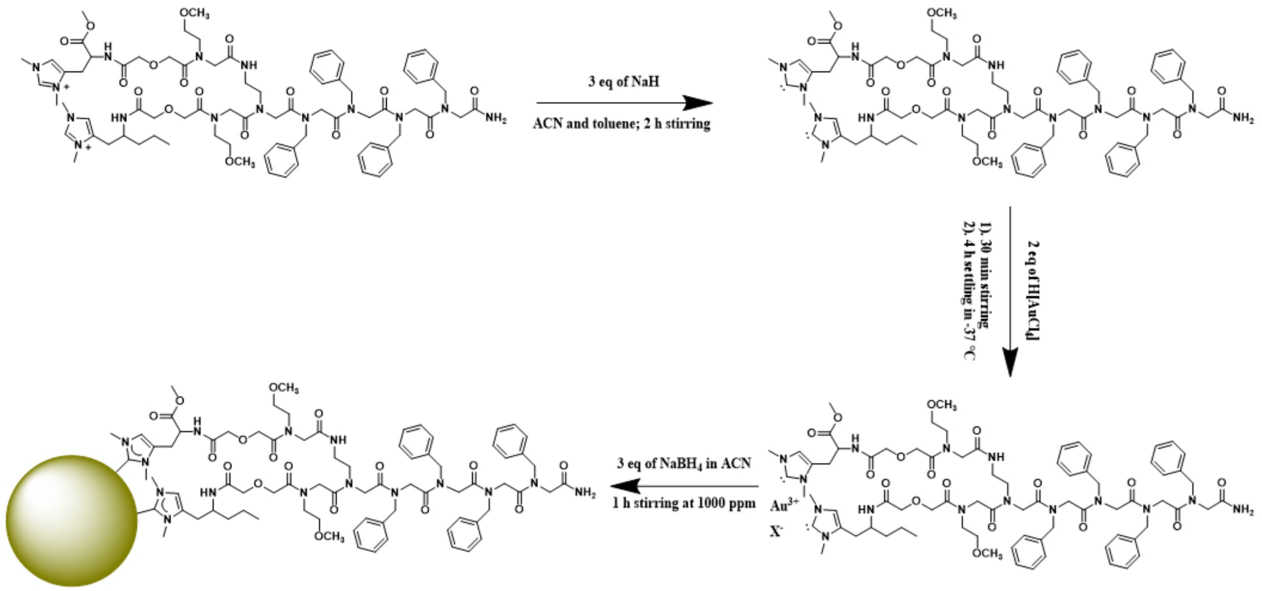

Chart S1. Synthesis procedure of peptoid-NHC@AuNPs (**Pep1** as an example)

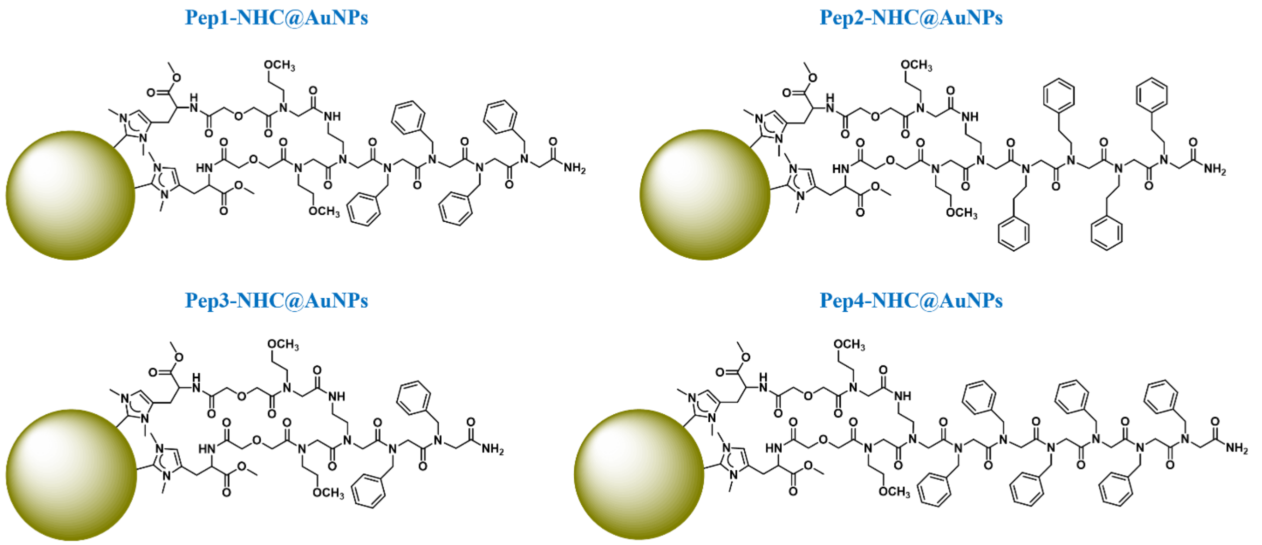

Chart S2. Chemical structure of peptoid-NHC@AuNPs

9. **Table of binding energy analysis for C 1s and N 1s HR-XPS scanning of Pep1, Pep1-NHC@AuNPs, Pep2, Pep2-NHC@AuNPs.**

Table S1. Binding energy of N 1s HR-XPS spectra of Pep1, Pep1-NHC@AuNPs, Pep2, Pep2-NHC@AuNPs.

Name of Samples	Pep1		
Bond Type	C=N–C	C–N–C	—NH_2
Binding Energy (eV)	401.78	400.16	399.51
**Name of Samples**	**Pep1-NHC@AuNPs**		
Bond Type	C=N–C	C–N–C	—NH_2
Binding Energy (eV)	400.68	399.88	398.88
**Name of Samples**	**Pep2**		
Bond Type	C=N–C	C–N–C	—NH_2
Binding Energy (eV)	401.88	400.26	399.48
**Name of Samples**	**Pep2-NHC@AuNPs**		
Bond Type	C=N–C	C–N–C	—NH_2
Binding Energy (eV)	400.81	399.90	398.92

Table S2. Binding energy of C 1s HR-XPS spectra of Pep1, Pep1-NHC@AuNPs, Pep2, Pep2-NHC@AuNPs.

Name of Samples	Pep1			
Bond Type	C–C<br>C=C	C–N<br>C–O	C=N<br>C=O	π–π* shake-up
Binding Energy (eV)	284.79	286.34	288.06	292.52
**Name of Samples**	**Pep1-NHC@AuNPs**			
Bond Type	C–C<br>C=C	C–N<br>C–O	C=N<br>C=O	C–Au
Binding Energy (eV)	284.80	286.1	287.88	284.23
**Name of Samples**	**Pep2**			
Bond Type	C–C<br>C=C	C–N<br>C–O	C=N<br>C=O	π–π* shake-up
Binding Energy (eV)	284.82	286.41	288.1	291.71
**Name of Samples**	**Pep2-NHC@AuNPs**			
Bond Type	C–C<br>C=C	C–N<br>C–O	C=N<br>C=O	C–Au
Binding Energy (eV)	284.83	286.08	287.96	284.3

10. **Size counts of Pep1-NHC@AuNPs vesicles**

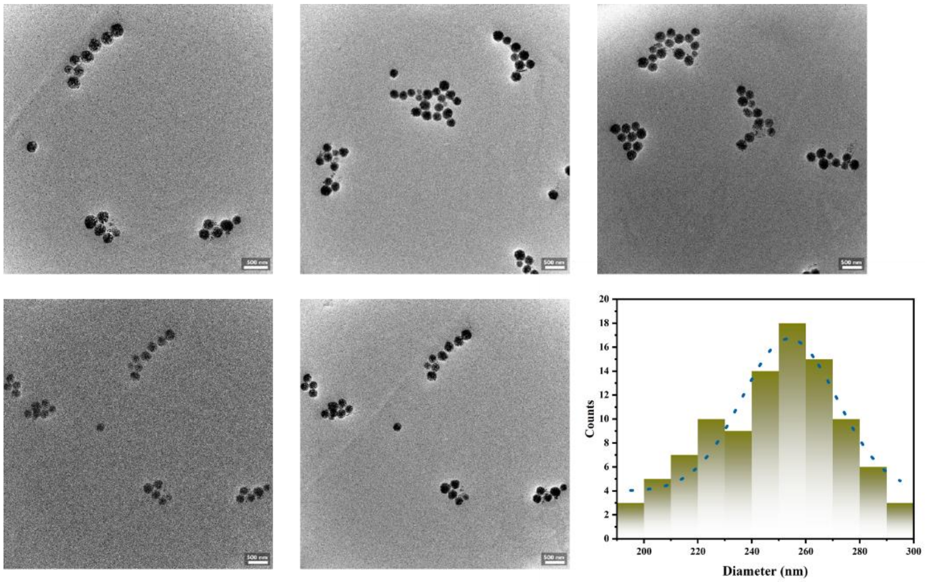

Figure S8. Size counts of Pep1-NHC@AuNPs vesicles with an average size of 248.1±24.54 nm

11. **Size counts of Pep2-NHC@AuNPs vesicles**

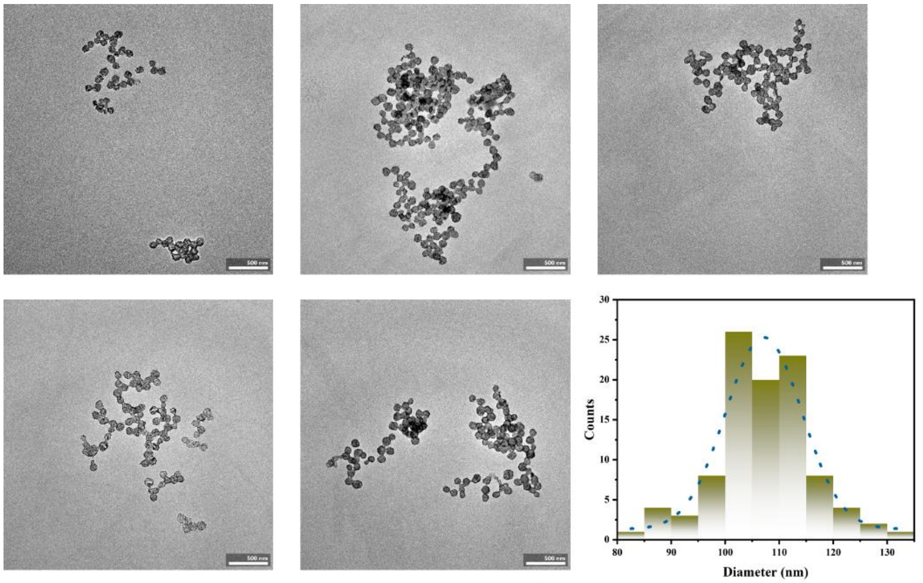

Figure S9. Size counts of Pep2-NHC@AuNPs vesicles with an average size of 107.1±9.04 nm

12. **Size counts of AuNPs in Pep1-NHC@AuNPs vesicles**

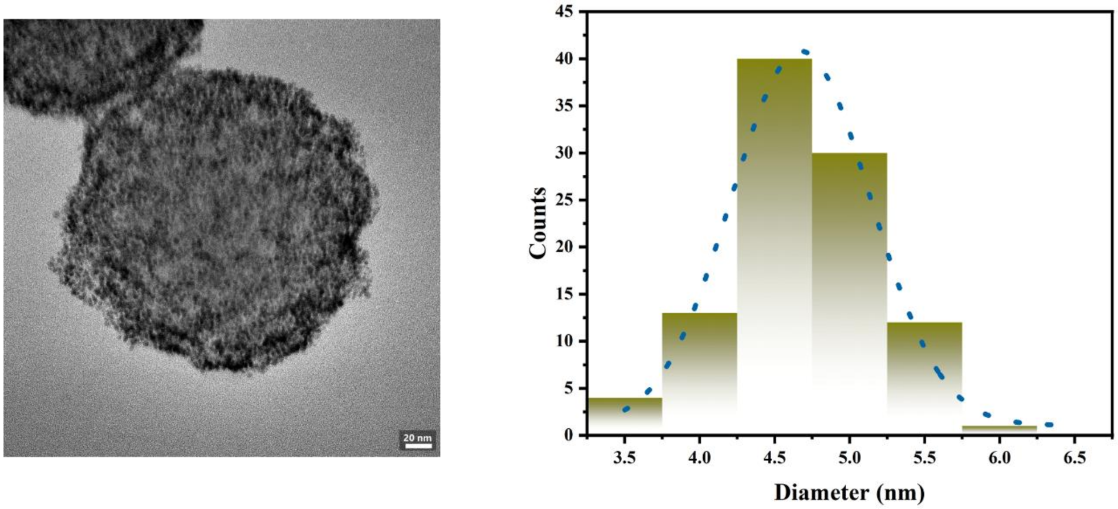

Figure S10. Size counts of AuNPs in Pep1-NHC@AuNPs vesicles with an average size of 4.7±0.50 nm

13. **Size counts of AuNPs in Pep2-NHC@AuNPs vesicles**

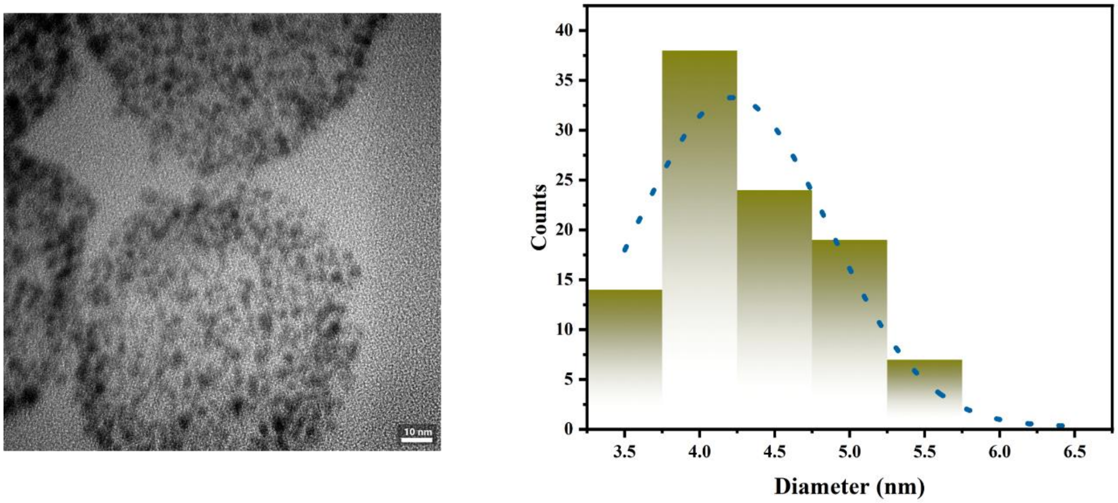

Figure S11. Size counts of AuNPs in Pep2-NHC@AuNPs vesicles with an average size of 4.3±0.51 nm

14. **Selected area electron diffraction (SAED) result of Peptoid-NHC@AuNPs vesicles**

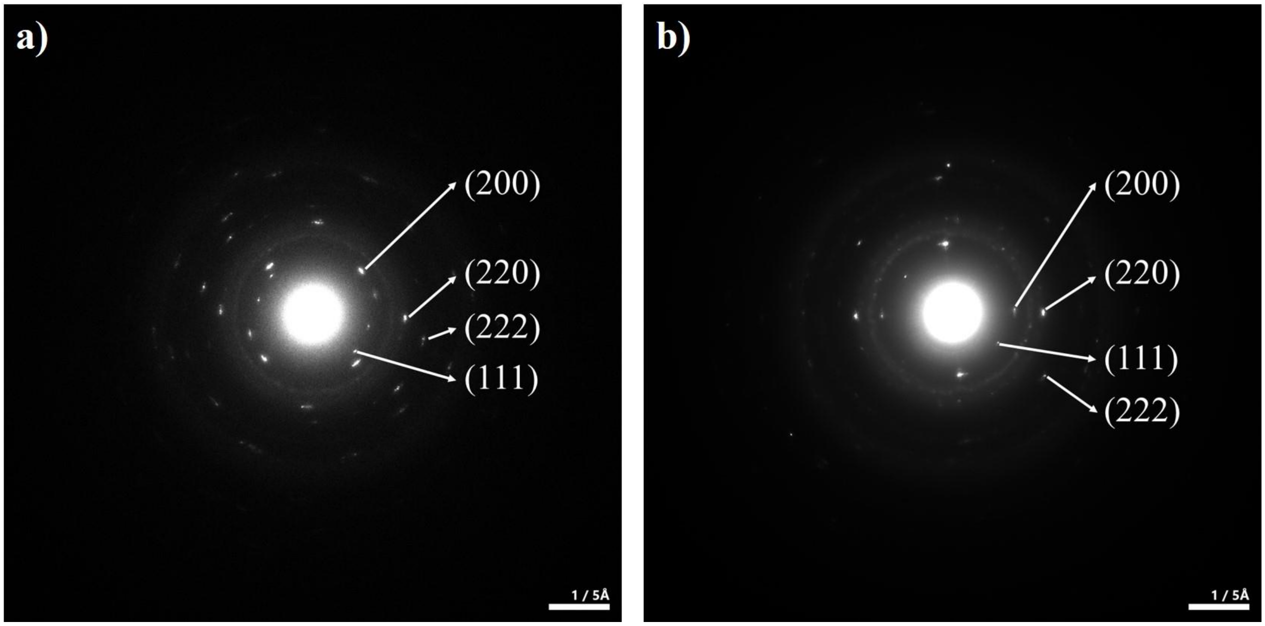

Figure S12. (a) SAED patterns of Pep1-NHC@AuNPs vesicles. (b) SAED patterns of Pep2-NHC@AuNPs vesicles.

15. **HAADF-STEM and 3D structure reconstruction of Pep2-NHC@AuNPs**

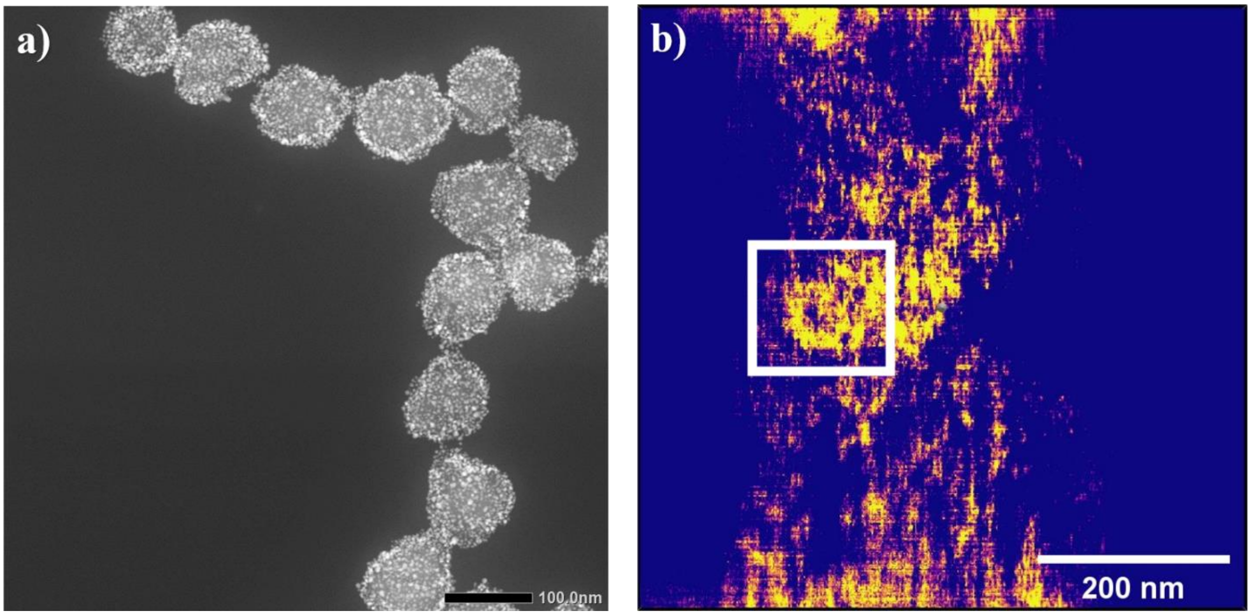

Figure S13. (a) HAADF-STEM image of Pep2-NHC@AuNPs. (b) 3D structure reconstruction of Pep2-NHC@AuNPs (The vesicle structure is observed in the white box).

16. **Synthesis results of peptoids and AuNPs nanohybrids without NaH**

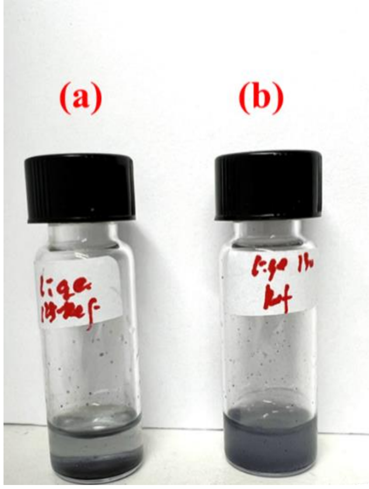

Figure S14. Synthesis results of peptoids and AuNPs nanohybrids without NaH: (a) Pep1; (b) Pep2.

17. DLS measurement for CMC calculation of Pep1-NHC@AuNPs

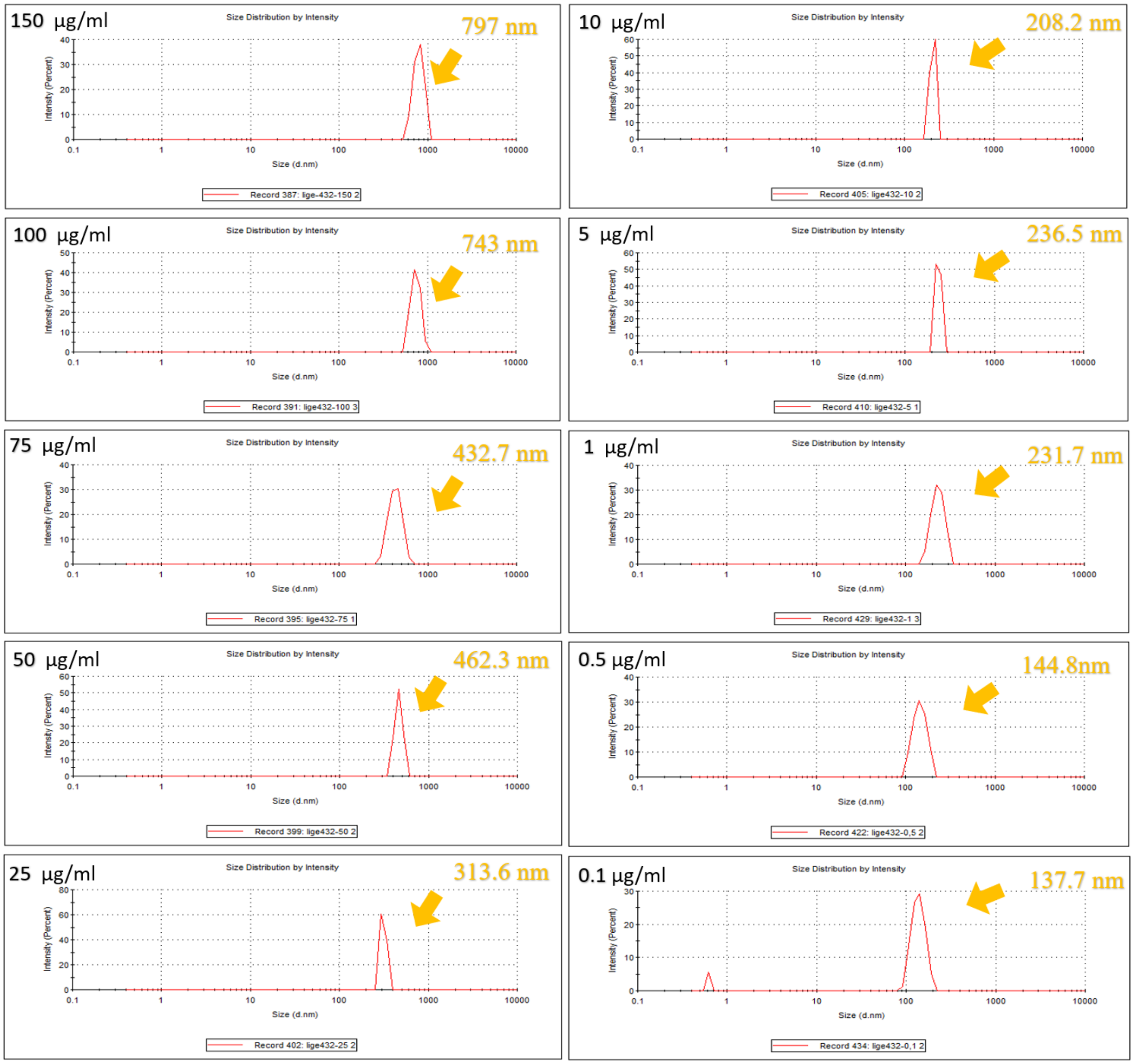

Figure S15. DLS measurement results for CMC calculation of Pep1-NHC@AuNPs

18. **DLS measurement for CMC calculation of Pep2-NHC@AuNPs**

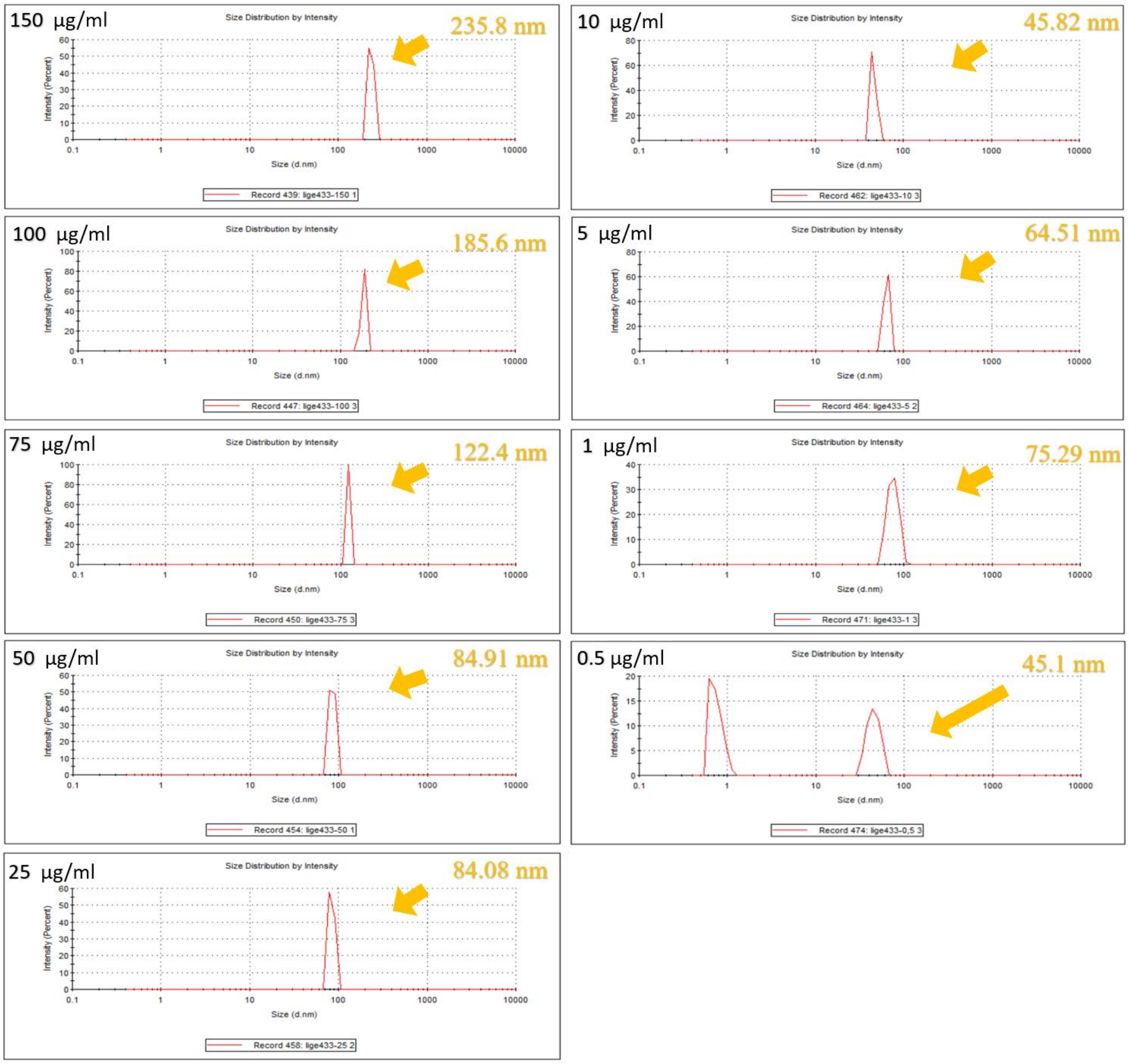

Figure S16. DLS measurement results for CMC calculation of Pep2-NHC@AuNPs

19. **Size counts of AuNPs in peptoid-NHC@AuNPs vesicles used for size comparison**

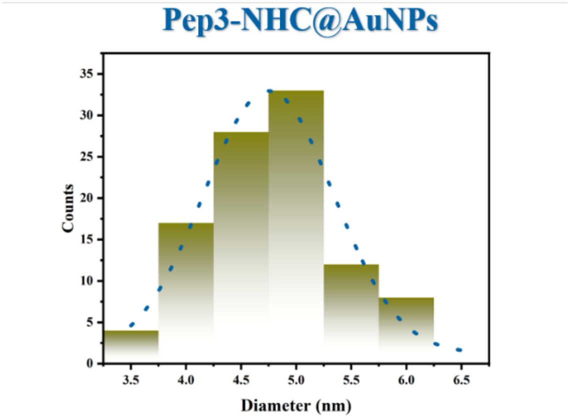

4.8 ± 0.59 nm

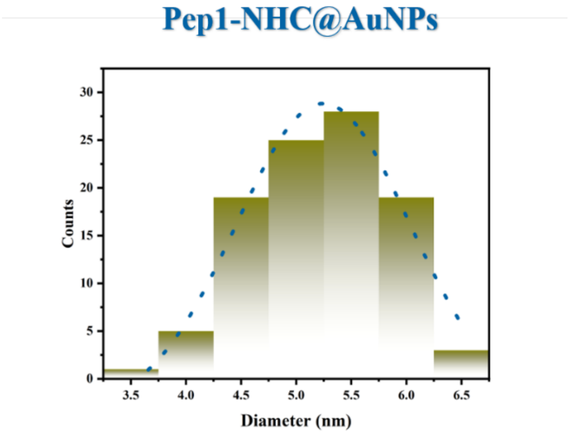

5.2 ± 0.62 nm

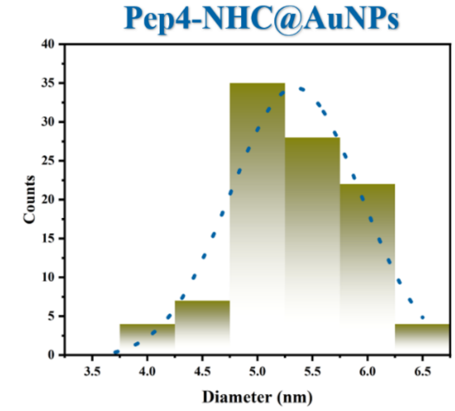

5.4 ± 0.55 nm

Figure S17. Size counts of AuNPs in peptoid-NHC@AuNPs vesicles used for size comparison

20. N 1s X-ray photoelectron spectra of peptoid-NHC@AuNPs for size comparison

Table S3. Binding energy of N 1s HR-XPS spectra of Pep1-NHC@AuNPs, Pep3-NHC@AuNPs, Pep4-NHC@AuNPs for size comparison.

Name of Samples	Pep1-NHC@AuNPs		
Bond Type	C=N−C	C−N−C	—NH_2
Binding Energy (eV)	400.68	399.2	397.73
Name of Samples	Pep3-NHC@AuNPs		
Bond Type	C=N−C	C−N−C	—NH_2
Binding Energy (eV)	400.59	399.4	398.34
Name of Samples	Pep4-NHC@AuNPs		
Bond Type	C=N−C	C−N−C	—NH_2
Binding Energy (eV)	400.28	398.7	396.54

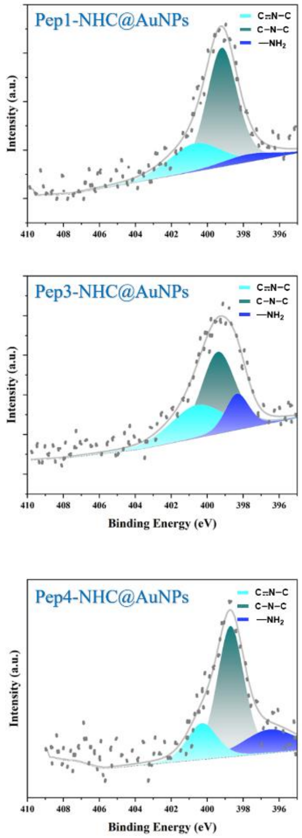

Figure S18. N 1s HR-XPS spectra of Pep1-NHC@AuNPs, Pep3-NHC@AuNPs, Pep4-NHC@AuNPs for size comparison.

21. C 1s X-ray photoelectron spectra of peptoid-NHC@AuNPs for size comparison

Table S4. Binding energy of C 1s HR-XPS spectra of Pep1-NHC@AuNPS, Pep3-NHC@AuNPs, Pep4-NHC@AuNPs for size comparison.

Name of Samples	Pep1-NHC@AuNPs			
Bond Type	C–C<br>C=C	C–N<br>C–O	C=N<br>C=O	C–Au
Binding Energy (eV)	284.83	285.35	286.10	284.25
Name of Samples	Pep3-NHC@AuNPs			
Bond Type	C–C<br>C=C	C–N<br>C–O	C=N<br>C=O	C–Au
Binding Energy (eV)	284.84	285.35	287.20	284.01
Name of Samples	Pep4-NHC@AuNPs			
Bond Type	C–C<br>C=C	C–N<br>C–O	C=N<br>C=O	C–Au
Binding Energy (eV)	284.78	285.35	288.16	284.06

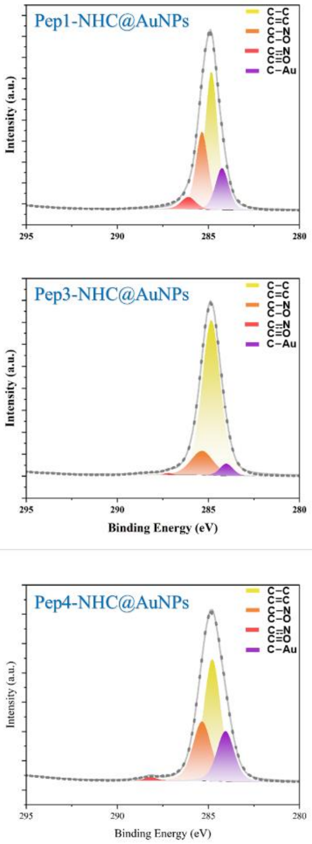

Figure S19. C 1s HR-XPS spectra of Pep1-NHC@AuNPS, Pep3-NHC@AuNPs, Pep4-NHC@AuNPs for size comparison.

22. **Size counts of Pep1-NHC@AuNPs vesicles for size comparison**

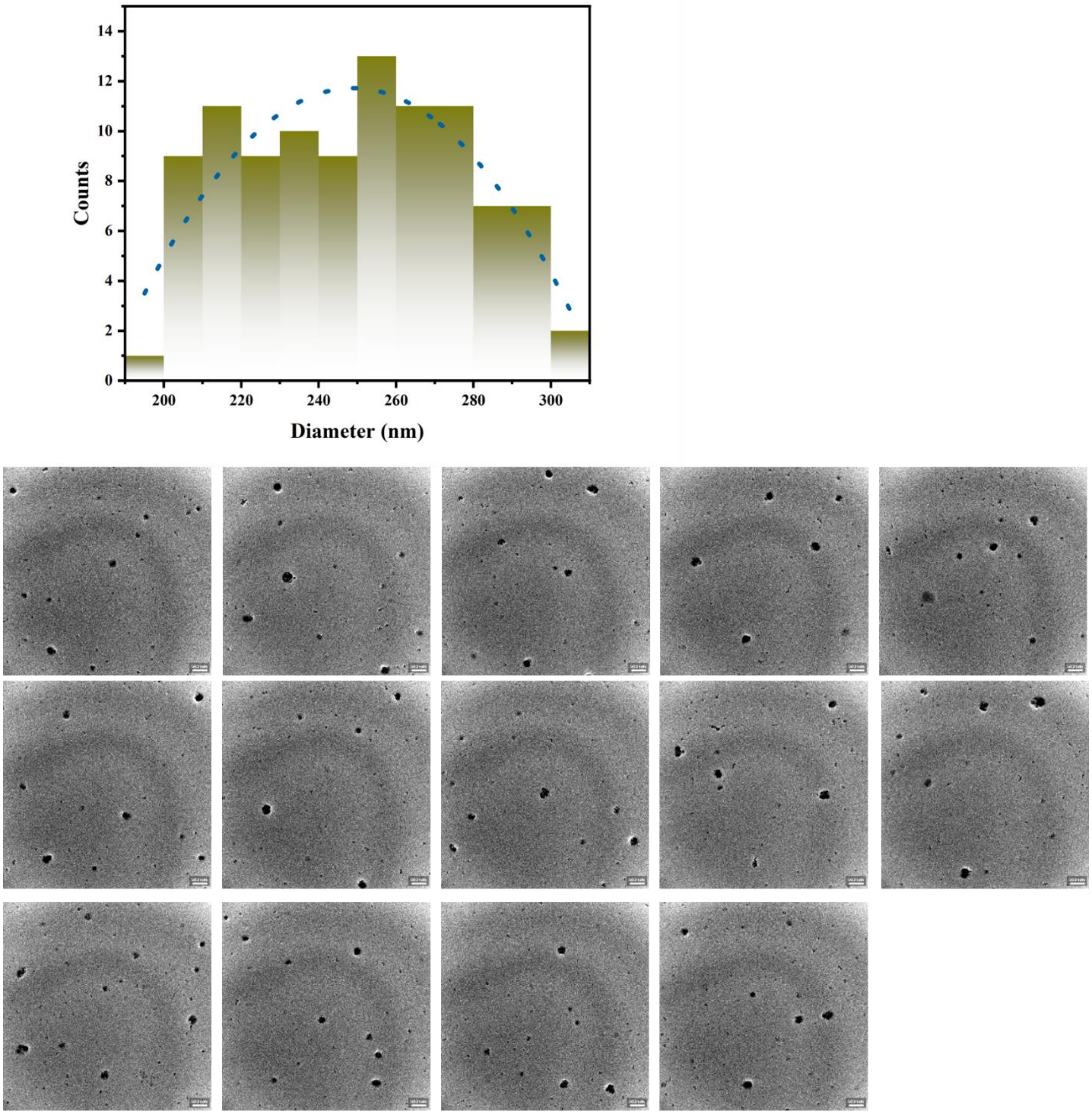

Figure S20. Size counts of Pep1-NHC@AuNPS vesicles for size comparison with an average size of 249.5±28.53 nm.

23. Size counts of Pep3-NHC@AuNPs for size comparison

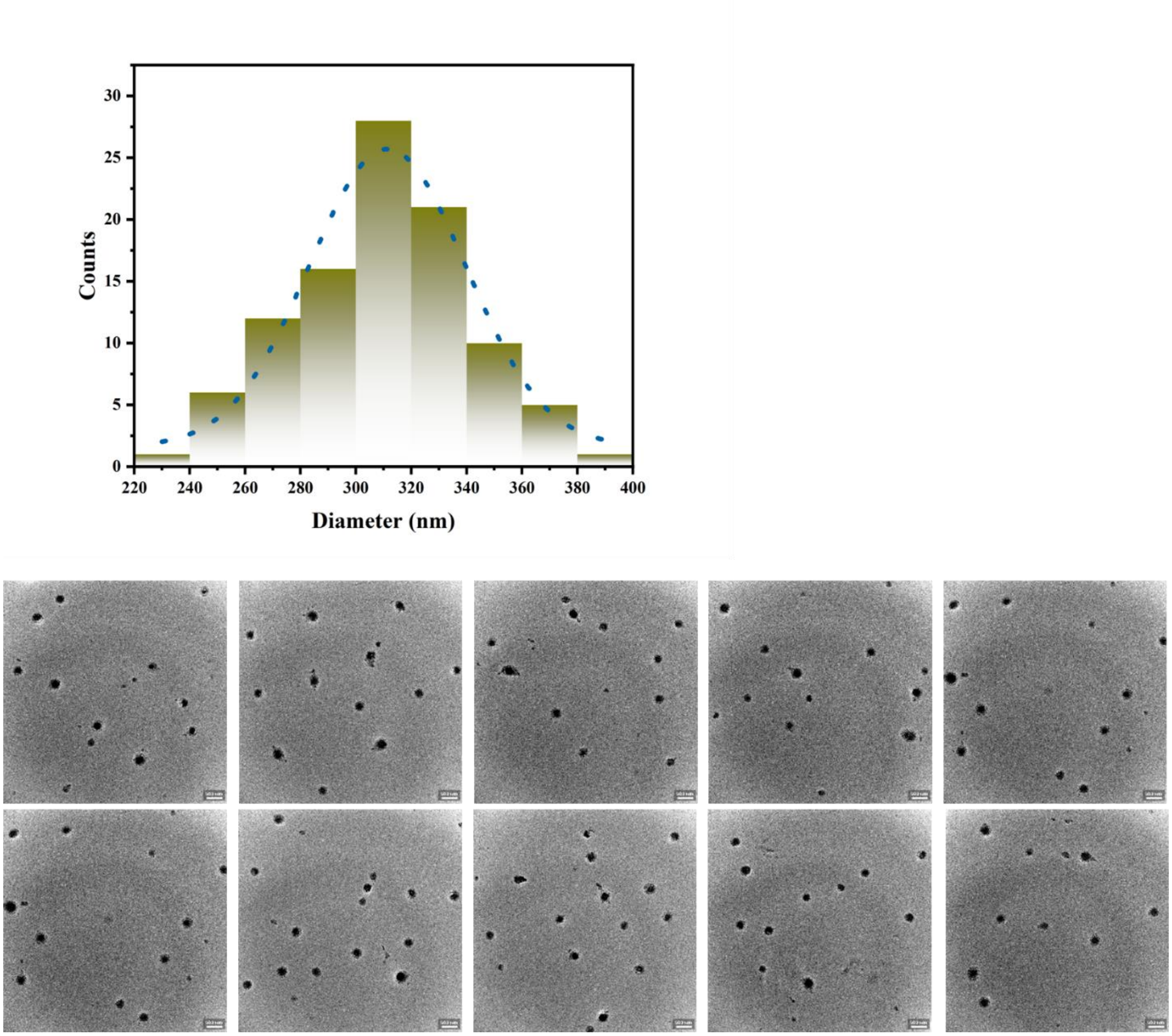

Figure S21. Size counts of Pep3-NHC@AuNPS vesicles for size comparison with average size of 308.3±31.08 nm.

24. Size counts of Pep4-NHC@AuNPs for size comparison

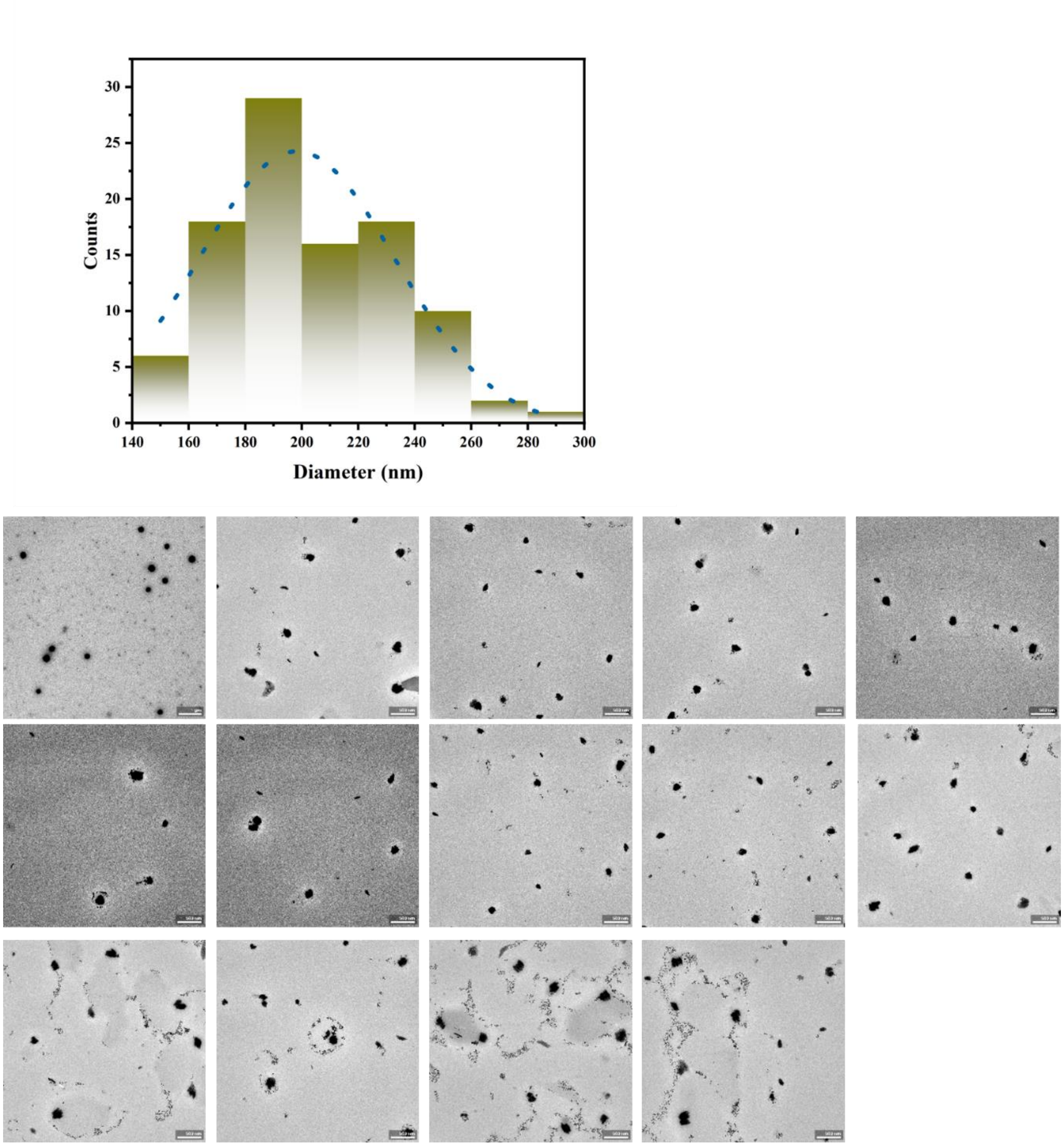

Figure S22. Size counts of Pep4-NHC@AuNPS vesicles for size comparison with average size of 203.3±30.56 nm.

25. **DLS Analysis of Pep1-NHC@AuNPS, Pep3-NHC@AuNPs, Pep4-NHC@AuNPs for size comparison.**

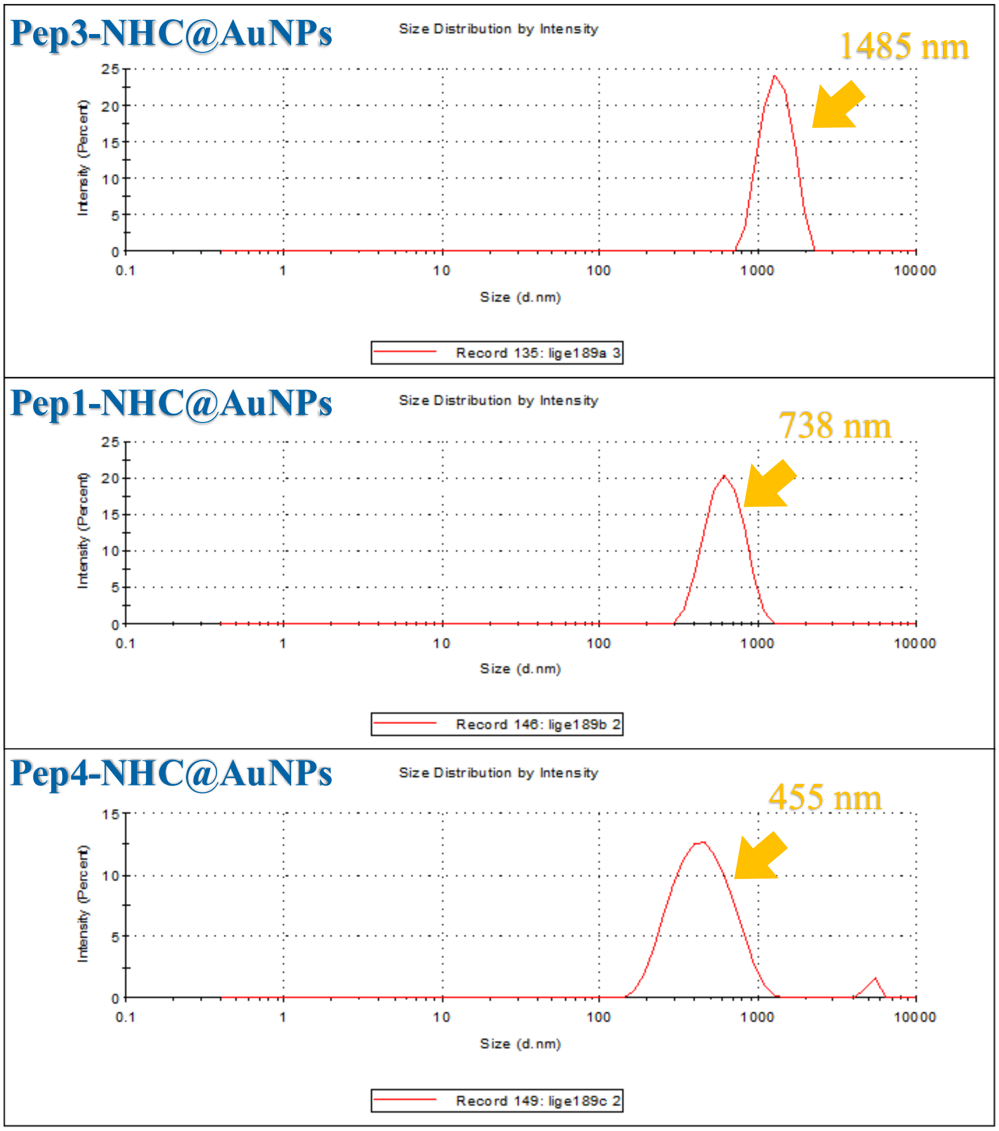

Figure S23. DLS Analysis of Pep1-NHC@AuNPS, Pep3-NHC@AuNPs, and Pep4-NHC@AuNPs for size comparison. When only 2 Npm groups are incorporated, an average DLS size of 1485 nm is observed. Notably, an increase in the number of Npm groups to 4 correlates with a reduction in DLS size to 738 nm. The smallest vesicle size was consistently observed when 6 Npm groups were present, resulting in a DLS size of 455 nm for Pep4-NHC@AuNPs.

26. UV-Vis absorption spectroscopy of Pep4-NHC@AuNPs in toluene, chloroform and water

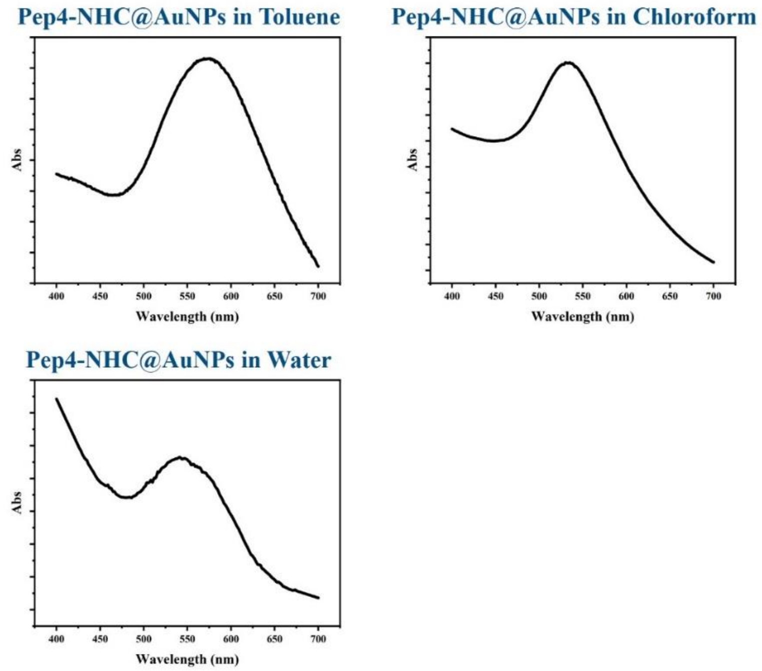

Figure S24. UV-Vis absorption spectroscopy of Pep4-NHC@AuNPs in toluene, chloroform and water.

27. TEM images of Pep4-NHC@AuNPs in toluene, chloroform and water

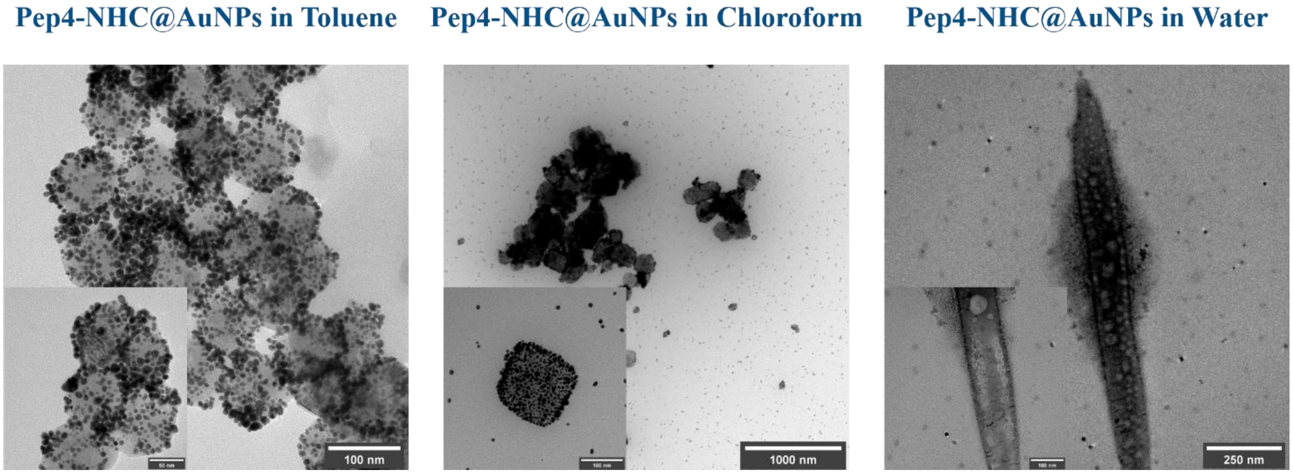

Figure S25. TEM images of Pep4-NHC@AuNPs in toluene, chloroform and water.

28. **X-ray diffraction (XRD) analysis of Pep4 assembly**

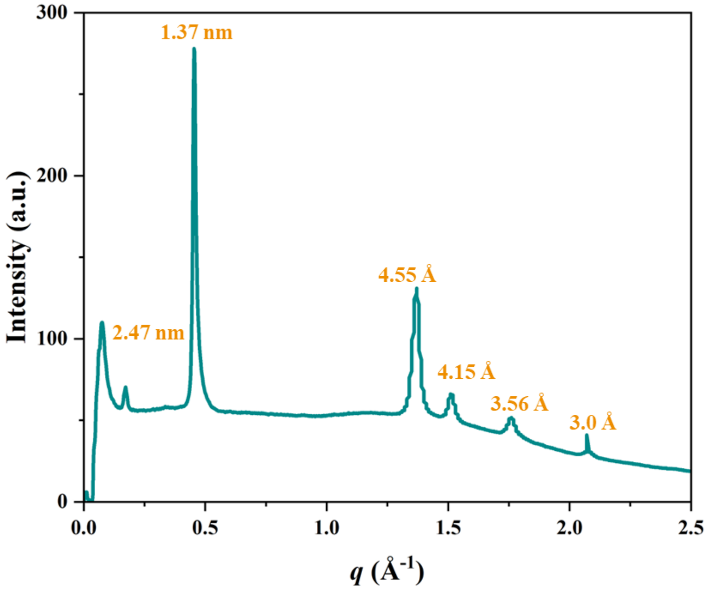

Figure S26. XRD profile of Pep4 assembly; the d spacing values are determined following the formula of d=2π/q.

29. **Synthesis and chemical structure of peptoid-S@AuNPs**

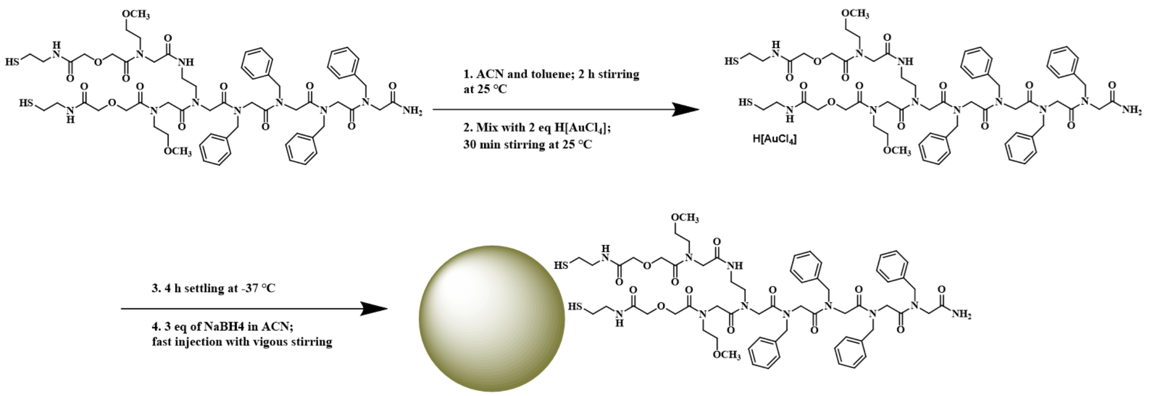

Chart S3. Synthesis procedure of peptoid-S@AuNPs (Pep5 as an example).

30. **UV-Vis absorption spectroscopy of Pep5-S@AuNPs**

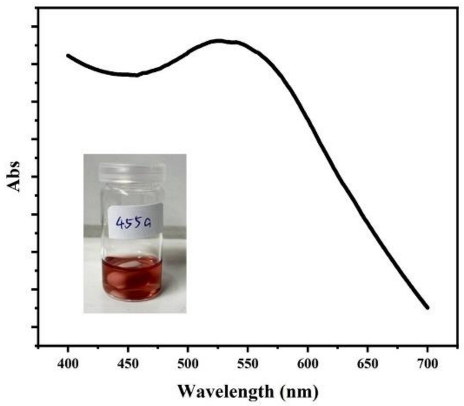

Figure S27. UV-Vis absorption spectroscopy of Pep5-S@AuNPs in toluene.

31. **XPS analysis of Pep5-S@AuNPs**

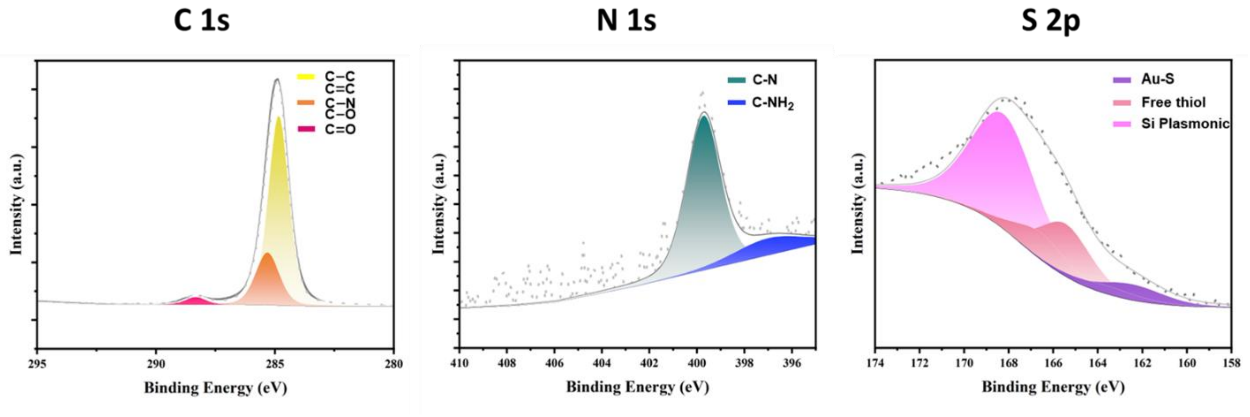

Figure S28. C 1S, N 1s, S 2p scanning of Pep5-S@AuNPs.

Table S5. Binding energy of C 1s HR-XPS spectra of Pep5-S@AuNPs.

**Name of Samples**	**Pep5-S@AuNPs**		
**Bond Type**	C–C<br>C=C	C–N<br>C–O	C=O
**Binding Energy (eV)**	284.84	285.31	288.33

Table S6. Binding energy of N 1s HR-XPS spectra of Pep5-S@AuNPs.

**Name of Samples**	**Pep5-S@AuNPs**	
**Bond Type**	C–N	—NH2
**Binding Energy (eV)**	399.72	396.99

Table S7. Binding energy of S 2p HR-XPS spectra of Pep5-S@AuNPs.

**Name of Samples**	**Pep5-S@AuNPs**		
**Bond Type**	Au-S	Free thiol	Si Plasmonic
**Binding Energy (eV)**	162.49	165.45	168.24

32. STEM measurement and STEM-EDS Mapping result of Pep5-S@AuNPs

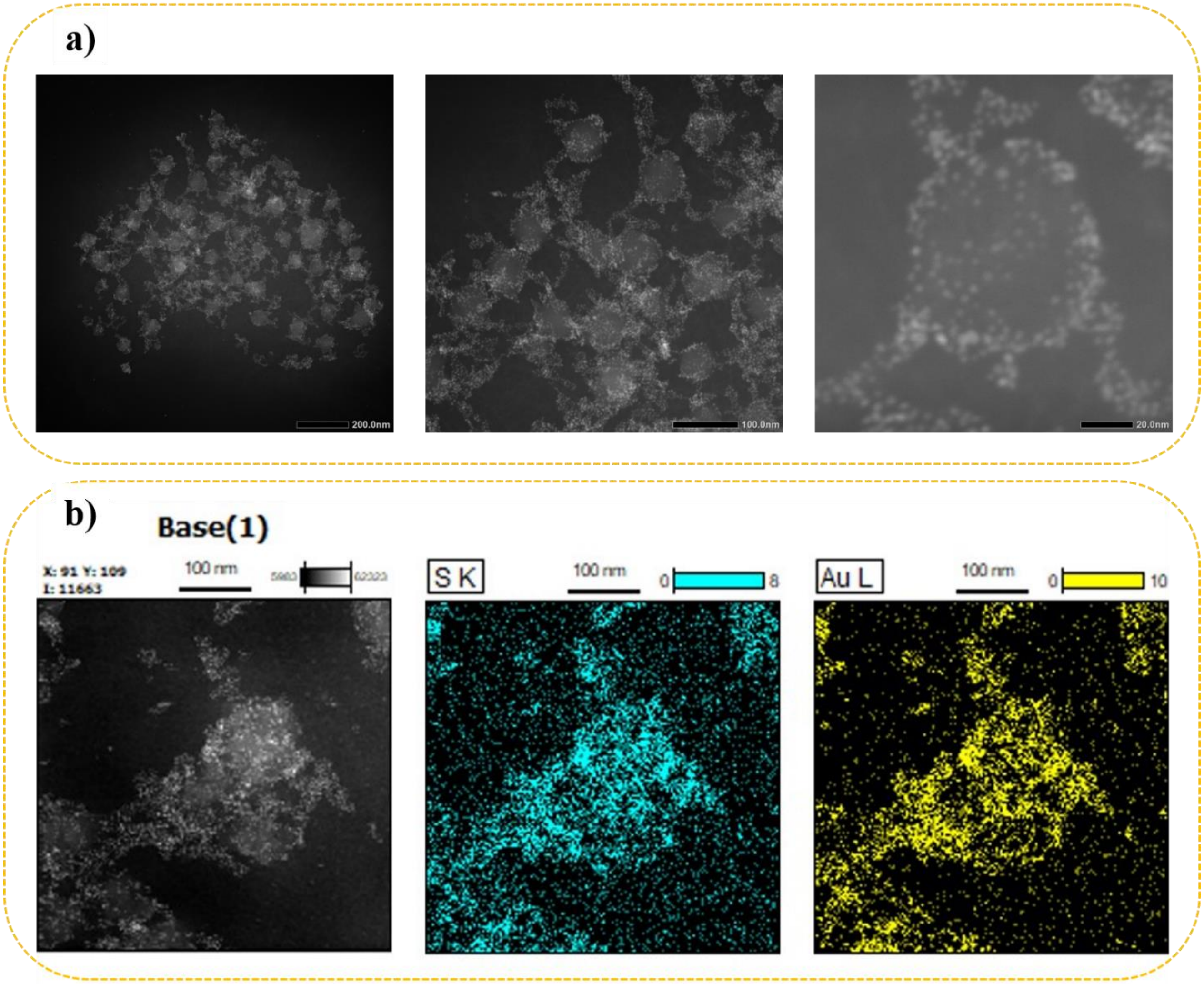

Figure S29. (a) STEM images of Pep5-S@AuNPs. (b) STEM-EDS Mapping result of Pep5-S@AuNPs.

33. **DLS measurements for thiol etching of Pep1-NHC@AuNPs over 24 h**

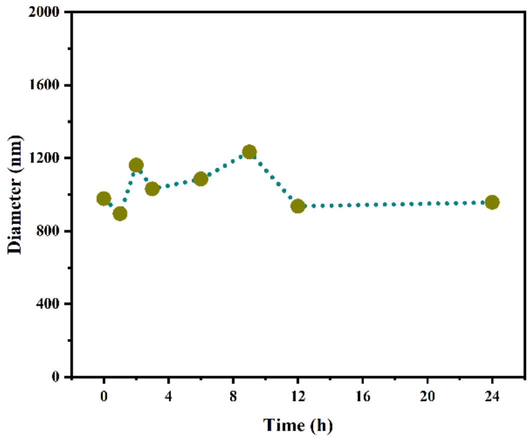

Figure S30. Stability test of peptoid-NHC@AuNPs vesicles: DLS analysis result of Pep1-NHC@AuNPs over thiol etching experiments.

34. **DLS measurement for thiol etching of Pep5-S@AuNPs over 24 h**

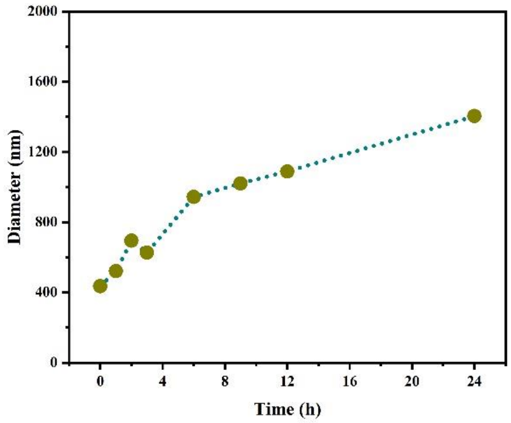

Figure S31. Stability test of peptoid-S@AuNPs vesicles: DLS analysis result of Pep5-S@AuNPs over thiol etching experiments.

35. DLS measurement results for thiol etching of Pep1-NHC@AuNPs over 24 h

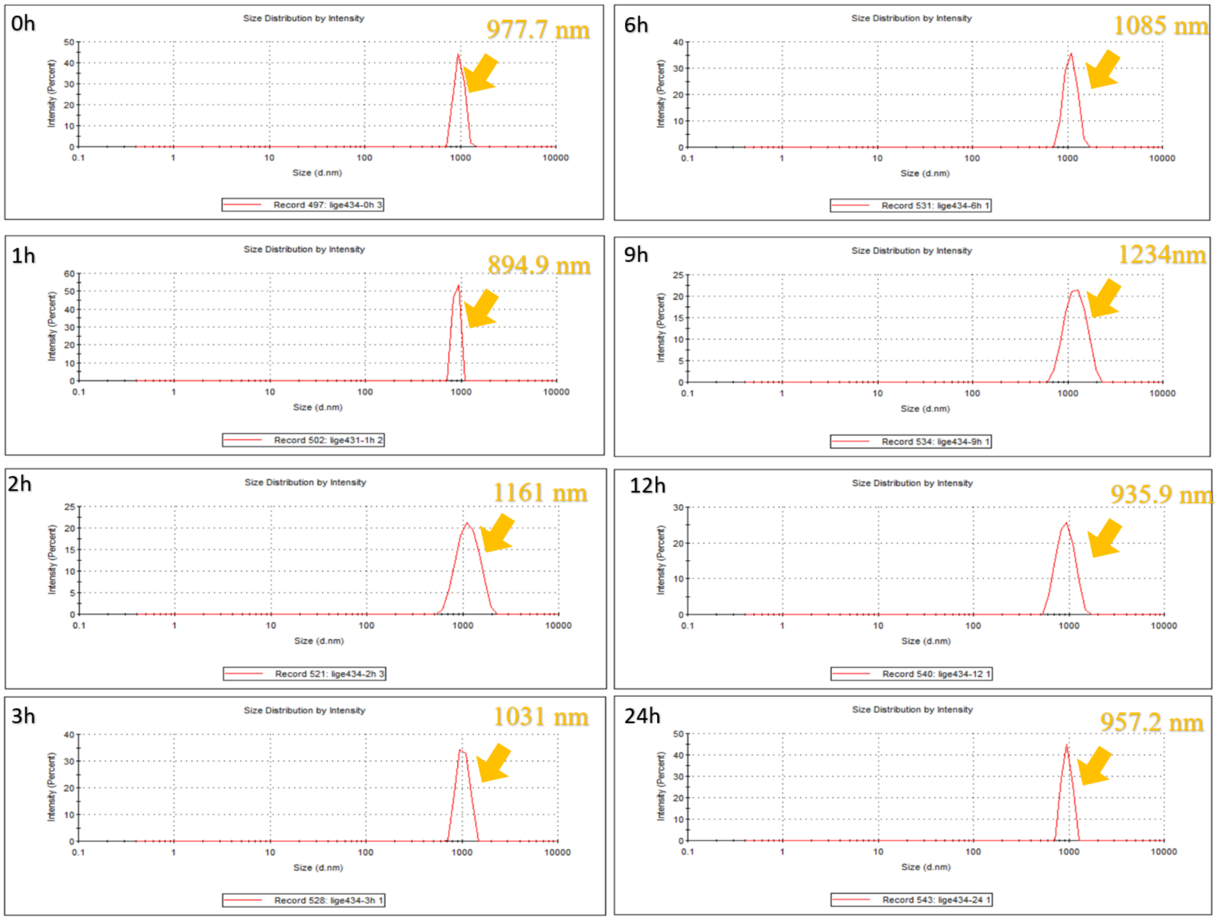

Figure S32. DLS measurement results of Pep1-NHC@AuNPs mixed with 0.5 mM 1-dodecanethiol at 0h, 1h, 2h, 3h, 6h, 9h, 12h, 24h in toluene.

36. DLS measurement results for thiol etching of Pep5-S@AuNPs over 24 h

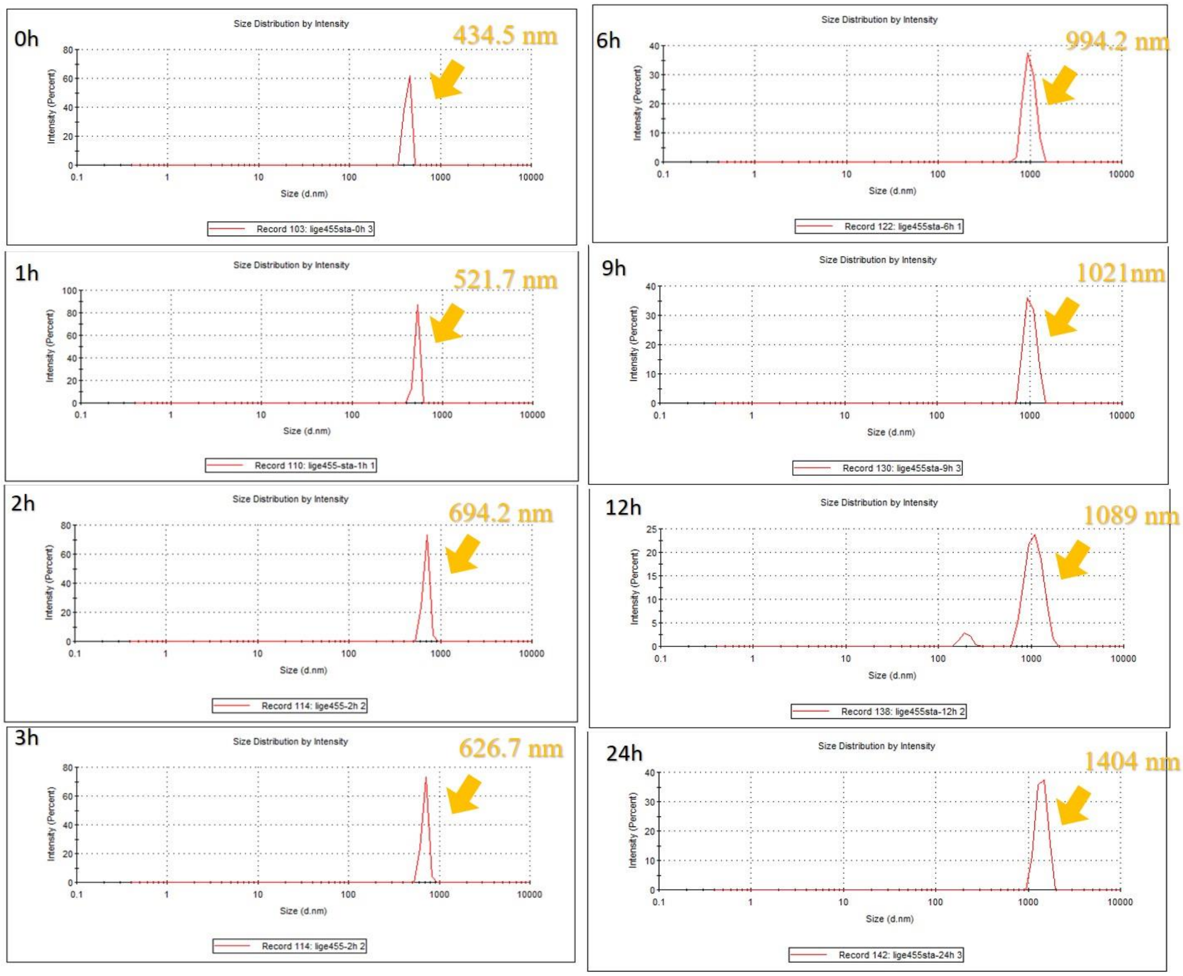

Figure S33. DLS measurement results of Pep5-S@AuNPs mixed with 0.5 mM 1-dodecanethiol at 0 h, 1 h, 2 h, 3 h, 6 h, 9 h, 12 h, 24 h in toluene.

37. **TEM and STEM images of Pep1-NHC@AuNPs after 24 h thiol etching**

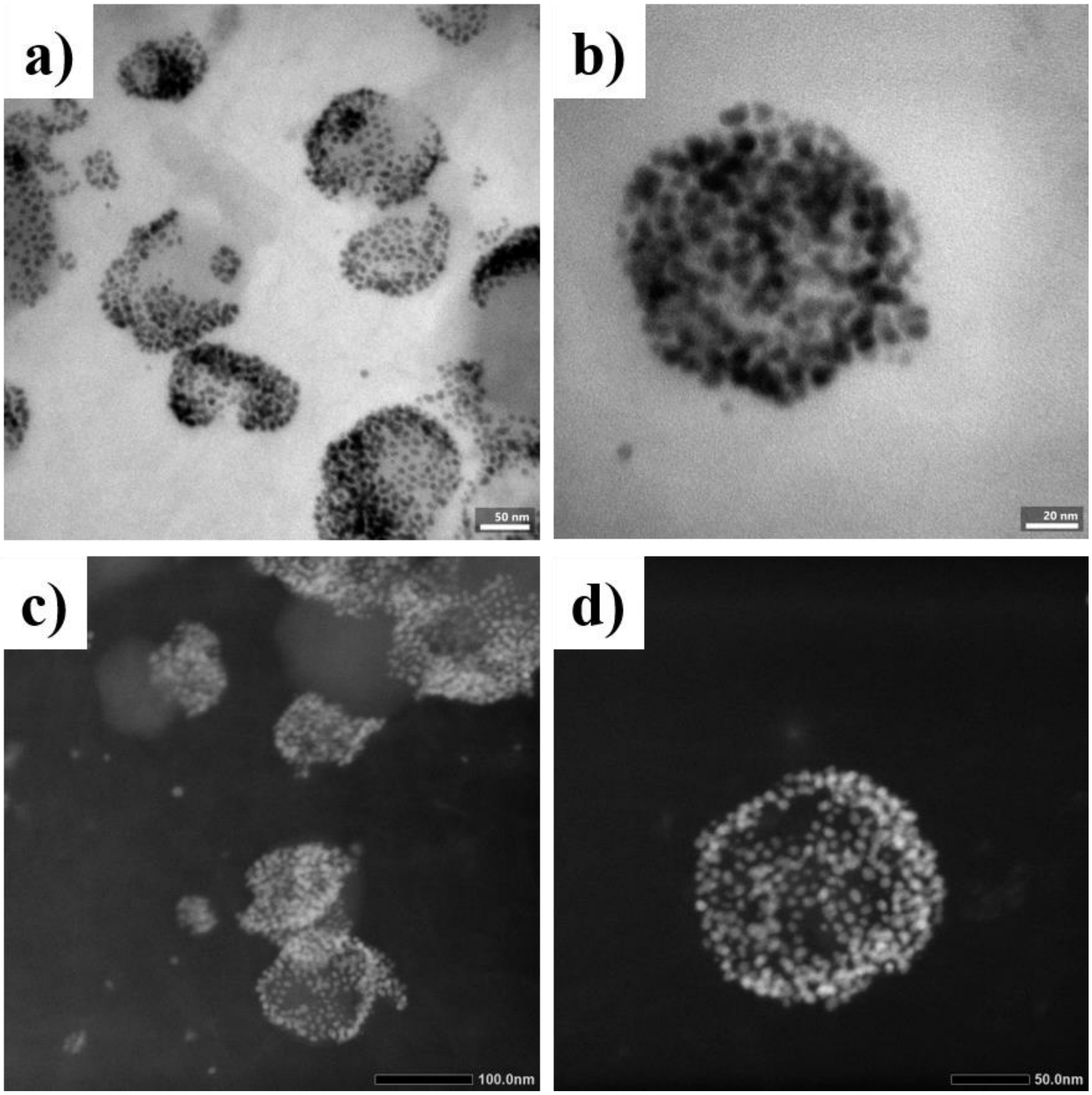

Figure S34. TEM (a ~ b) and STEM (c ~ d) images of Pep1-NHC@AuNPs after 24 h thiol etching.

38. **TEM and STEM images of Pep5-S@AuNPs after 24 h thiol etching**

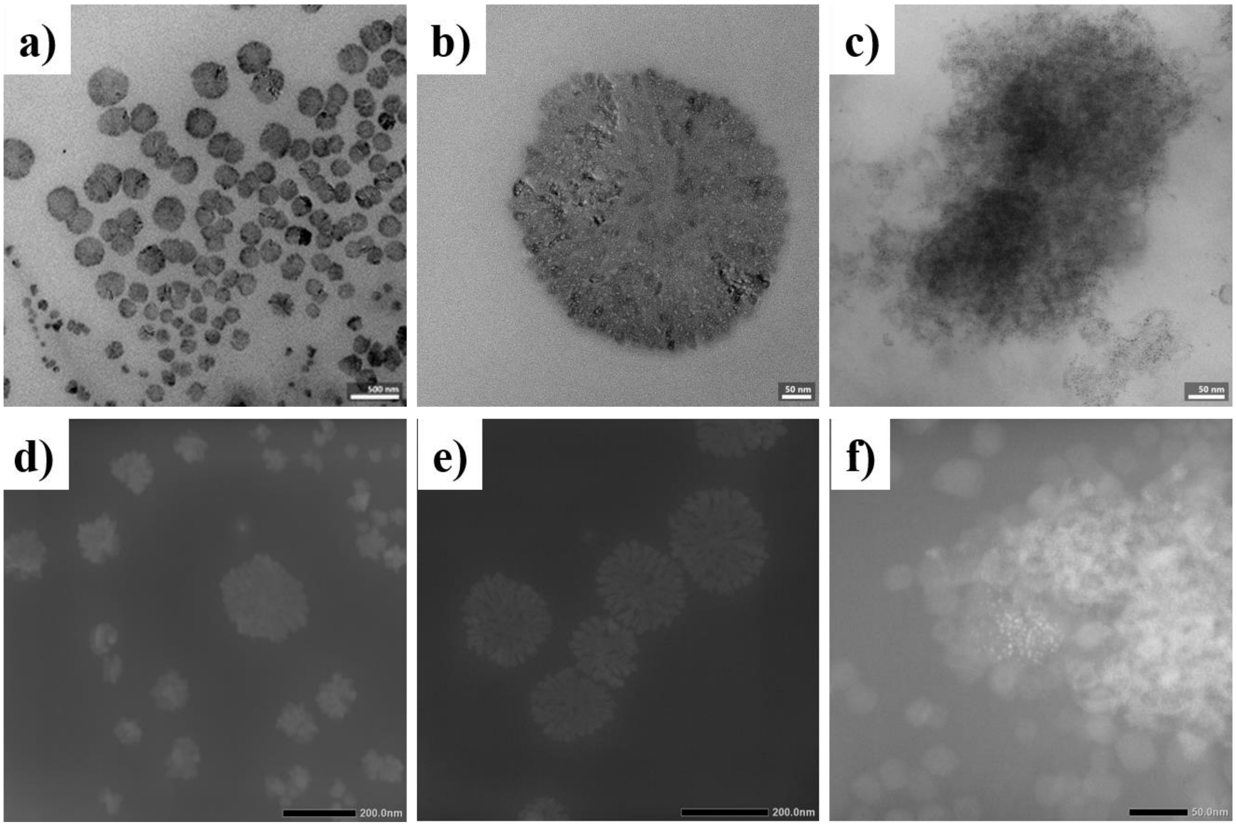

Figure S35. TEM (a ~ c) and STEM (d ~ f) images of Pep5-S@AuNPs after 24 h thiol etching.

39. **STEM-EDS measurement of Pep5-S@AuNPs after 24 h thiol etching**

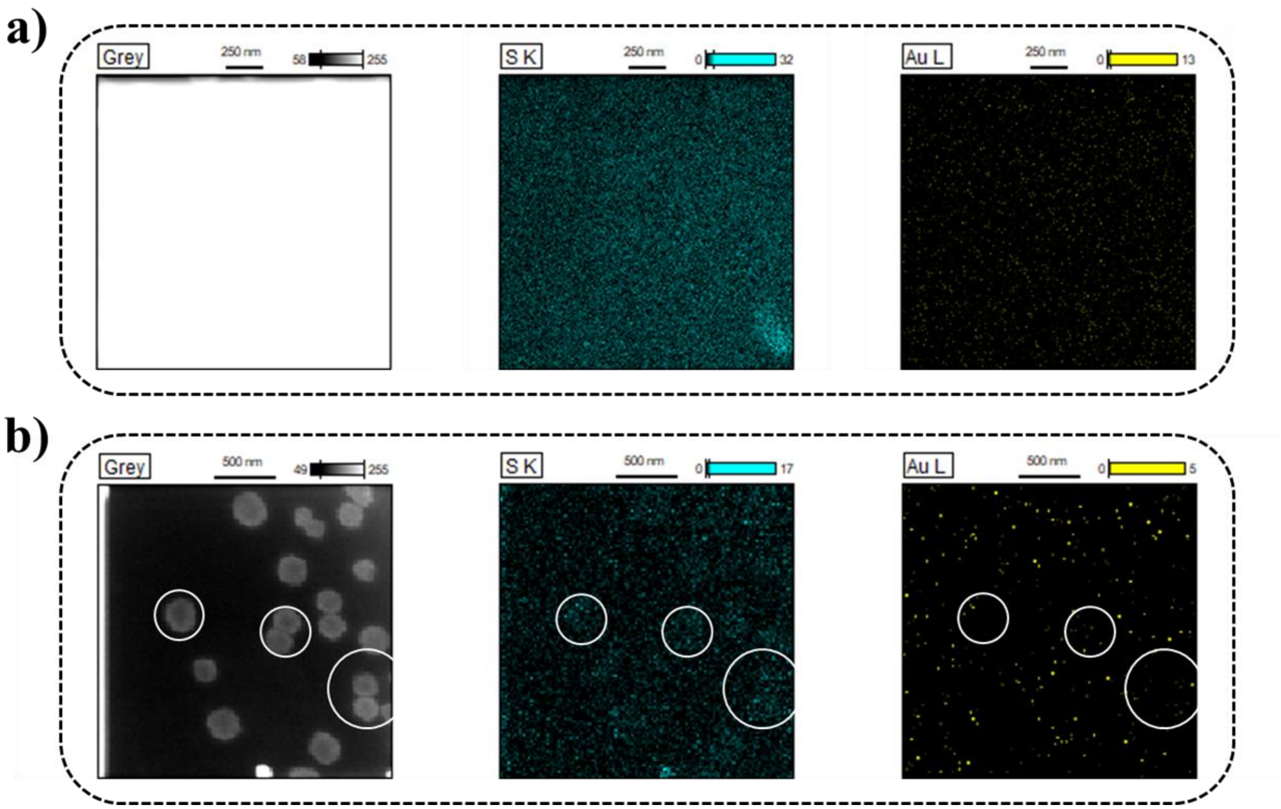

Figure S36. STEM-EDS measurement of Pep5-S@AuNPs after 24 h thiol etching: (a) shows that due to the lack of AuNPs, the pure organic assemblies burned after a 6 min scan; we therefore performed STEM-EDS measurements based on a fast scan (1 second) rate with 50 times stacks: the distribution of S is recognizable, but Au is difficult to identify.

40. **C 1s and N 1s X-ray photoelectron spectra of Pep1-NHC@AuNPs after thiol etching**

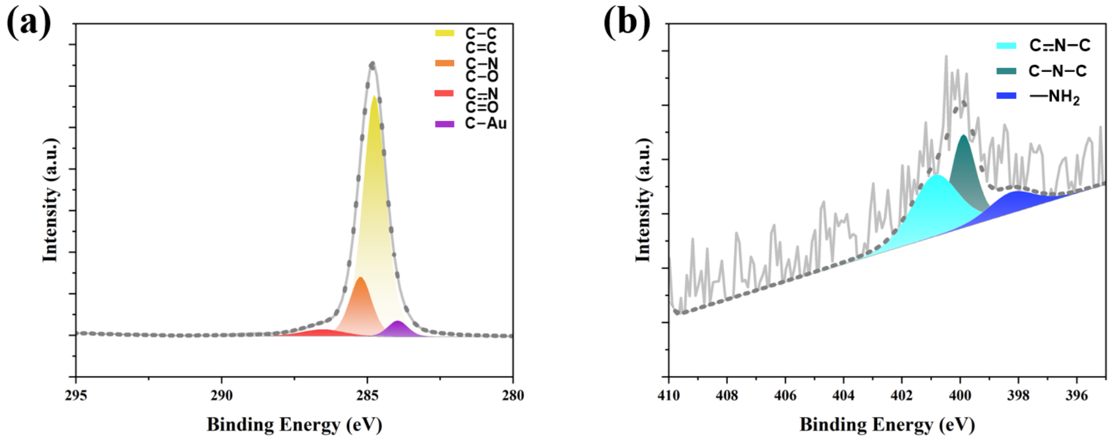

Figure S37. (a) C 1s HR-XPS spectrum of Pep1-NHC@AuNPs after thiol etching. (b) N 1s HR-XPS spectrum of Pep1-NHC@AuNPs after thiol etching.

Table S8. Binding energy of C 1s HR-XPS spectrum of Pep1-NHC@AuNPs after thiol etching.

Name of Samples	Pep1-NHC@AuNPs after thiol etching			
Bond Type	C–C Aliphatic	C–N Aliphatic<br>C–O Aliphatic	C=O Aliphatic<br>C=C Aromatic	C–Au
Binding Energy (eV)	284.76	285.23	286.53	283.96

Table S9. Binding energy of N 1s HR-XPS spectrum of Pep1-NHC@AuNPs after thiol etching.

Name of Samples	Pep1-NHC@AuNPs after thiol etching		
Bond Type	C=N–C	N–C=O–C	—NH_2
Binding Energy (eV)	400.87	399.90	398.25

41. **Microscope images of drop-casting result and selected Raman mapping area (within green square)**

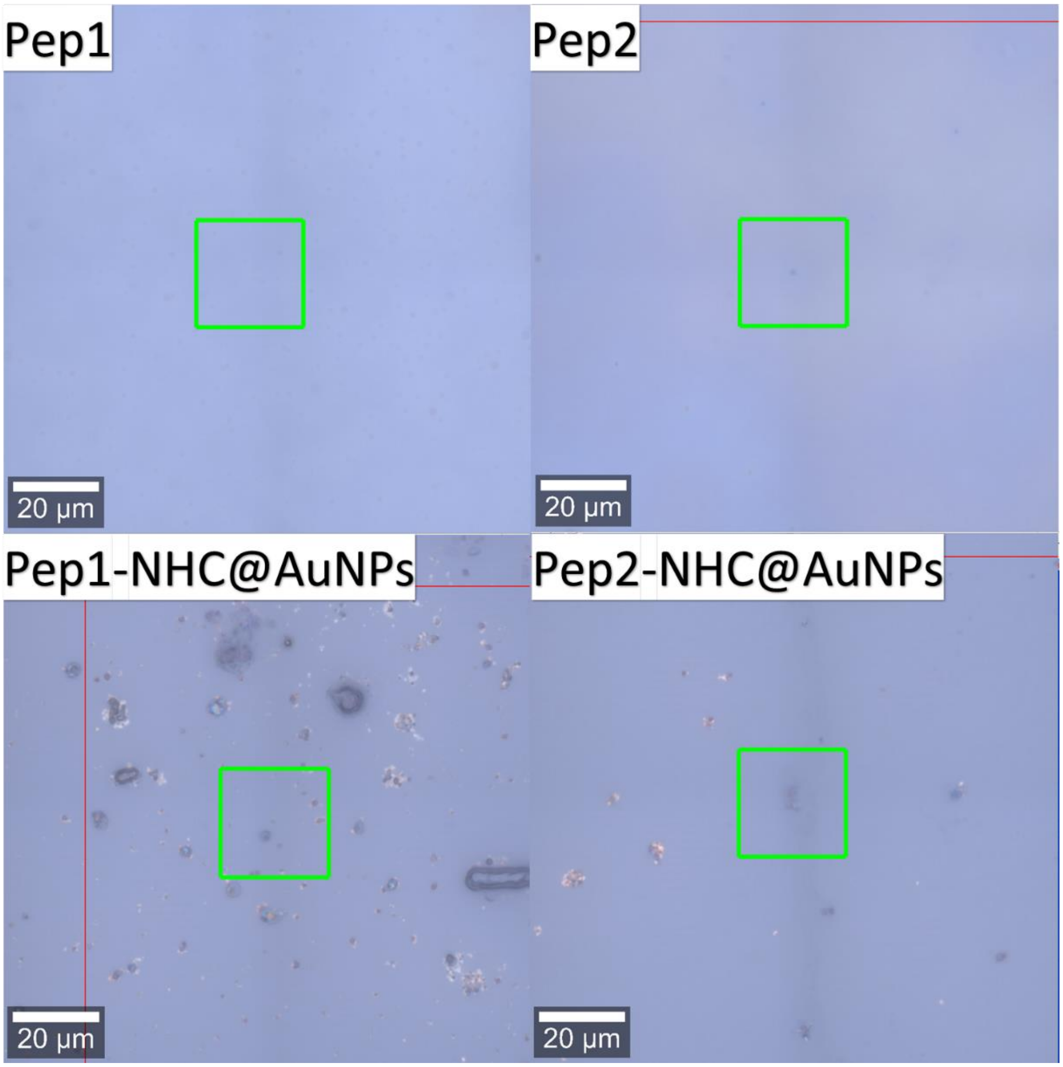

Figure S38. Microscope Images of drop-casting samples and selected Raman mapping area (within green square Pep1-NHC@AuNPS, Pep3-NHC@AuNPs, Pep4-NHC@AuNPs for size comparison).

42. **Raman measurement results of Pep1, Pep2, Pep1-NHC@AuNPs, and Pep2-NHC@AuNPs.**

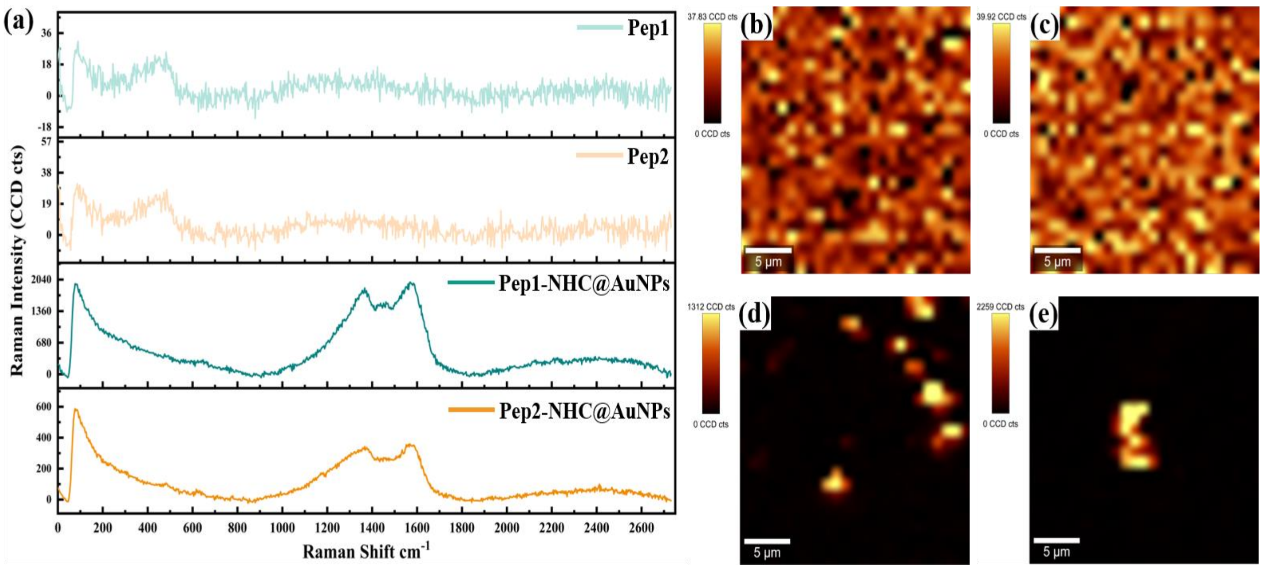

Figure S39. (a) Raman spectroscopy analysis of Pep1, Pep2, Pep1-NHC@AuNPs, and Pep2-NHC@AuNPs. (b) Raman mapping of Pep1. (c) Raman mapping of Pep2. (d) Raman mapping of Pep1-NHC@AuNPs. (e) Raman mapping of Pep2-NHC@AuNPs.

43. **Calculated SNR result of Pep1, Pep1-NHC@AuNPs, Pep2, Pep2-NHC@AuNPs.**

Table S10. SNR calculation result of Pep1, Pep1-NHC@AuNPs, Pep2, Pep2-NHC@AuNPs.

	Pep1	Pep2	Pep1-NHC@AuNPs	Pep2-NHC@AuNPs
RMS of 1500 ~ 1700 cm^{-1}	4.55	5.62	1419.19	249.52
RMS of 1800 ~ 2000 cm^{-1}	3.72	4.16	57.6	8.35
SNR	1.75	1.8	57.83	29.51

44. **Raman Measurement results of Pep5-S@AuNPs**

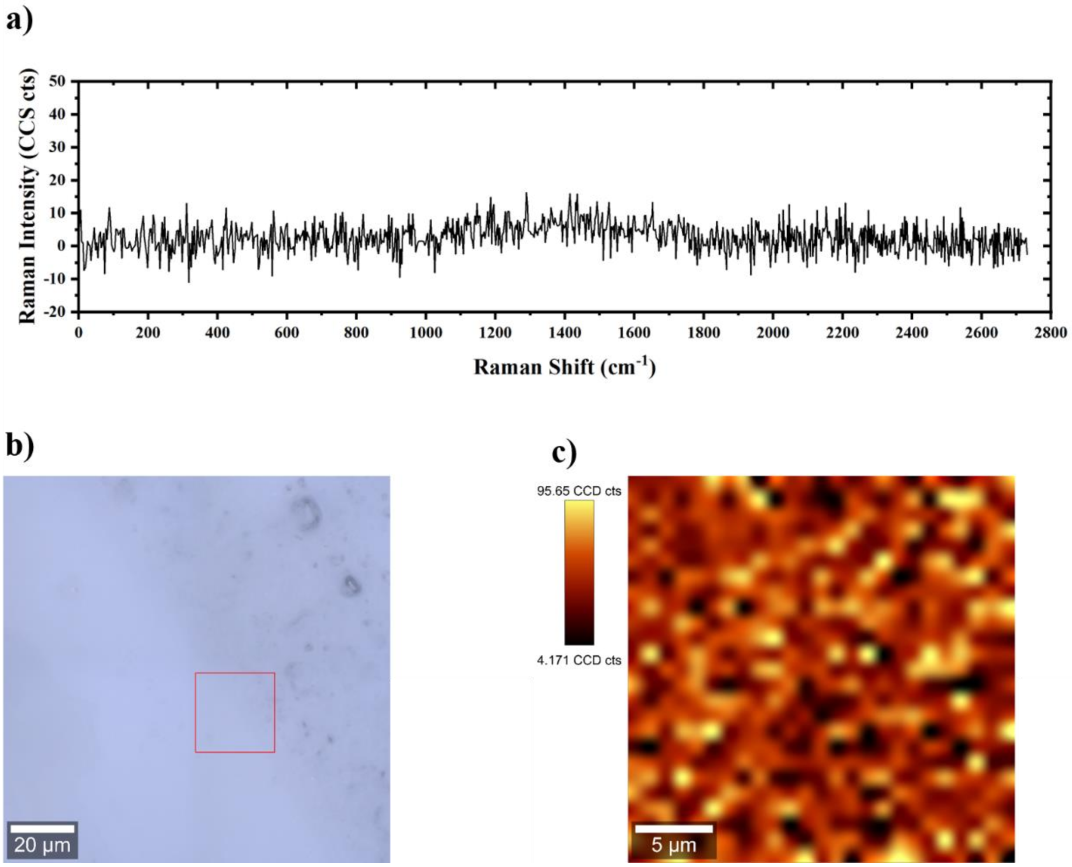

Figure S40. a) Raman spectroscopy analysis of Pep5-S@AuNPs. (b) Microscope Images of drop-casting samples of and selected Raman mapping area. (c) Raman mapping of Pep5-S@AuNPs.

45. **Calculated SNR result of Pep5-S@AuNPs.**

Table S11. SNR calculation result of Pep5-S@AuNPs.

	RMS of 1500 ~ 1700 cm^{-1}	RMS of 1800 ~ 2000 cm^{-1}	SNR
**Pep5-S@AuNPs**	2.67	1.87	3.08

46. Reference

(1) Ruan, C.-Y.; Murooka, Y.; Raman, R. K.; Murdick, R. A. Dynamics of Size-Selected Gold Nanoparticles Studied by Ultrafast Electron Nanocrystallography. *Nano Letters* **2007**, *7* (5), 1290-1296. DOI: 10.1021/nl070269h

(2) Jin, H.; Jiao, F.; Daily, M. D.; Chen, Y.; Yan, F.; Ding, Y.-H.; Zhang, X.; Robertson, E. J.; Baer, M. D.; Chen, C.-L. Highly stable and self-repairing membrane-mimetic 2D nanomaterials assembled from lipid-like peptoids. *Nature Communications* **2016**, *7* (1), 12252. DOI: 10.1038/ncomms12252

(3) Semmlow, J. *Signals and systems for bioengineers: a MATLAB-based introduction*; Academic press, 2011.

(4) Wang, M.; Song, Y.; Zhang, S.; Zhang, X.; Cai, X.; Lin, Y.; Yoreo, J. J. D.; Chen, C.-L. Programmable two-dimensional nanocrystals assembled from POSS-containing peptoids as efficient artificial light-harvesting systems. *Science Advances* **2021**, *7* (20), eabg1448. DOI: doi:10.1126/sciadv.abg1448
